# L-arginine metabolism as pivotal interface of mutual host–microbe interactions in the gut

**DOI:** 10.1080/19490976.2023.2222961

**Published:** 2023-06-26

**Authors:** Björn Nüse, Tim Holland, Manfred Rauh, Roman G. Gerlach, Jochen Mattner

**Affiliations:** a Mikrobiologisches Institut - Klinische Mikrobiologie, Immunologie und Hygiene, Universitätsklinikum Erlangen and Friedrich-Alexander-Universität (FAU) Erlangen-Nürnberg, Erlangen, Germany; b Department of Pediatrics and Adolescent Medicine, Universitätsklinikum Erlangen and Friedrich-Alexander-Universität (FAU) Erlangen-Nürnberg, Erlangen, Germany; c Medical Immunology Campus Erlangen, FAUErlangen-Nürnberg, Erlangen, Germany

**Keywords:** L-arginine (L-arg), intestinal microbiota, mucosal immune function, microbial pathogenesis, mutual metabolic pathways, colonization resistance, host–microbe interaction, virulence factor, dietary L-arg supplementation

## Abstract

L-arginine (L-arg) is a versatile amino acid and a central intestinal metabolite in mammalian and microbial organisms. Thus, L-arg participates as precursor of multiple metabolic pathways in the regulation of cell division and growth. It also serves as a source of carbon, nitrogen, and energy or as a substrate for protein synthesis. Consequently, L-arg can simultaneously modify mammalian immune functions, intraluminal metabolism, intestinal microbiota, and microbial pathogenesis. While dietary intake, protein turnover or de novo synthesis usually supply L-arg in sufficient amounts, the expression of several key enzymes of L-arg metabolism can change rapidly and dramatically following inflammation, sepsis, or injury. Consequently, the availability of L-arg can be restricted due to increased catabolism, transforming L-arg into an essential amino acid. Here, we review the enzymatic pathways of L-arg metabolism in microbial and mammalian cells and their role in immune function, intraluminal metabolism, colonization resistance, and microbial pathogenesis in the gut.

## Introduction

L-arginine (L-arg) exhibits remarkable metabolic and regulatory versatility in and among many organisms. It occurs universally across all kingdoms of life and plays a key role in host–microbe interactions.^1^ Thus, L-arg metabolism is of central importance for multiple biological processes and mutual interactions of mammals, microbes, and plants.^2–6^

Furthermore, L-arg is a semi-essential or conditionally essential amino acid in many species.^7,8^ Thus, endogenous synthesis and dietary intake of L-arg usually meet the metabolic demands of the respective organism. However, in conditions of catabolic stress or intestinal-renal dysfunction, the interorgan axis is pivotal for endogenous L-arg synthesis,^9–11^ the levels of available L-arg might not sufficienly meet metabolic needs anymore.^12,13^ Accordingly, L-arg needs to be supplemented by diet.^7^

L-arg is also a central intestinal metabolite.^14^ Thus, L-arg serves as a substrate for intestinal and microbial cells that colonize the largest interface at which our body crosstalks to its microbiota. The mutual interactions between intestinal tissues and microbiota that might even outnumber our own cells play a pivotal role for the digestion and metabolism of ingested food particles, for the intestinal circulation and vessel permeability as well as for the control of inflammation and colonization resistance.^15,16^ Thus, intestinal microbiota signal to local and distant tissues of the host body and influence multiple physiologic interactions and pathophysiologic processes, on the one hand, .^17,18^ On the other hand, the immune system of the host prevents the translocation of harmful signals and substances from the gut into the tissue and balances the homeostasis between the internal and external environments of the intestinal tract.^19^

The human gut microbiota also modulates immunity through the release of ligands and metabolites that translocate from the gut into the local and systemic circulation.^20^ A disruption of the symbiotic interactions between intestinal tissues and the microbiota can simultaneously alter the immune response of the host and the composition of the intestinal microbiota. These alterations consequently lead to a disrupted intraluminal metabolism and an increased intestinal permeability and thus, can trigger the development of inflammatory or infectious disease. Intestinal dysbiosis and the leaky gut syndrome indeed accompany many immune-mediated disorders including inflammatory bowel disease (IBD).^21^ Alterations in microbial metabolite pools can even predict IBD types.^22,23^

Several mutual interactions between gut microbiota and body tissues also affect the metabolism of L-arg. Thus, a disrupted L-arg metabolism is associated with immune-mediated or infectious diseases.^24^ Intraluminal L-arg levels might not even sufficiently fulfill metabolic demands in both disorders.^14^ This is particularly important since the fractional synthesis rate of proteins within the intestinal metabolism depends on the accessibility of the metabolic precursor pool in the gut.^25^ This intraluminal synthesis rate is indeed higher than in other major metabolically active tissues, such as the liver or muscle.^14^ Infection- or inflammation-associated L-arg deficiency contributes to immunopathology (Table 1), and clinical trials involving L-arg supplementation have shown a substantial contribution of L-arg in decreasing complications of inflammation and infection.^64,65^ Actually, the depletion of L-arg is one of the strategies used throughout nature to control the growth of organisms competing for the same biological niche.^66^ Moreover, invading pathogens exploit L-arg starvation as a survival strategy.^34,66,67^

**Table 1.** Overview over selected diseases with disruptions of L-arg metabolism.

Disease	Alterations in L-arg metabolism and functional consequences	Ref
Sepsis	depletion of plasma L-arginine levels → systemic L-arg – deficiency	^13,26–28^
	→ decreased arginine : ornithine ratio/endothelial dysfunction, decreased NO production/availability	^29–33^
	→ dysregulated immune responses/immunosuppression → myeloid suppressor cell expansion, T cell exhaustion	^35–37^
	→ enhanced gut permeability and bacterial translocation	^38–39^
NEC	decreased plasma L-arg levels at time of diagnosis	^40–43^
	decreased expression of L-arg synthesizing enzymes; increased expression of catabolic enzymes	^44^
Infections	depletion of L-arg as immune evasion mechanism	^45–49^
	decrease availability of NO synthesis and increase ornithine production by increased ADI activity in bacteria	^45–50,51^
IBD	altered availability of L-arg in feces and serum → compartmentalized L-arg – deficiency	^52–56^
	→ alterations in microbiota composition and microbiota-mediated immune responses	^52–57,113^
	→ alterations in L-arg transport	^53–54,58^
	→ endothelial dysfunction	^52–59^
	→ decreased L-arg biosynthesis	^58^
Covid-19	arginine depletion → enhanced arginase activity and endothelial dysfunction, hypercoagulability, immune dysfunction	^60–63^

While studies have focussed preferentially on the immune defense of the host in the past, information on the influence of metabolic alterations in L-arg metabolism on the composition of the intestinal microbiota and on microbial pathogenesis are limited. The most exciting studies in this area over the past years indicated novel roles for L-arg – converting enzymes in health and disease, as a result of their effects to limit the availability of free L-arg and altering consequently intraluminal metabolism and the composition of intestinal microbiota. Furthermore, L-arg affects the stress response of pathogenic bacteria and parasites and the expression of virulence factors that we will review and discuss here as well.

## Mammalian L-arg metabolism

L-arg metabolism in mammals underlies the production of a diverse range of products, including agmatine, creatine, glutamate, homoarginine, nitric oxide (NO), polyamines, proline, and urea.^6^ Furthermore, L-arg is pivotal for the synthesis and the modification of proteins.^3^ Consequently, methylated arginines such as asymmetric (ADMA) or symmetric dimethylarginine (SDMA) can be released upon degradation of proteins.^3^ Interestingly, the combined ratios of ADMA, L-arg and SDMA as well as the availability of NO have been suggested as markers for endothelial dysfunction, oxidative stress, or survival of patients in a variety of disorders including IBD, necrotizing enterocolitis (NEC), a devastating intestinal disease affecting premature or very low birth weight infants, and sepsis.^13,26−28,43−56,68−71^ Thus, the bioavailability of L-arg and its metabolic products is critical for the maintenance of proper organ homeostasis.

**Figure 1.**
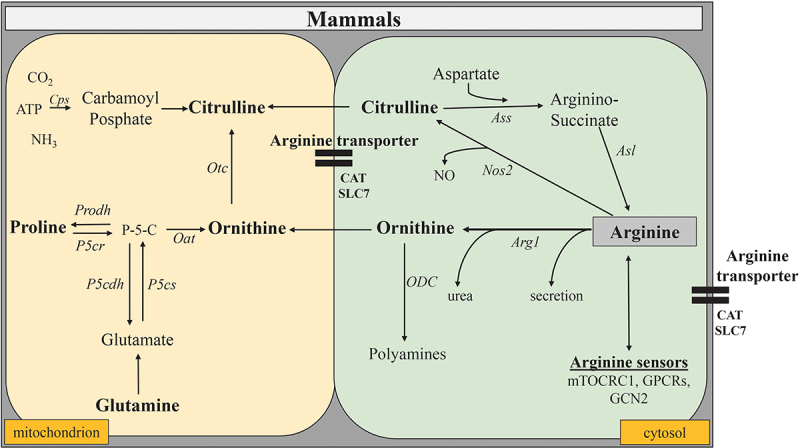
Anabolic and catabolic pathways of L-arg metabolism in mammalian cells. The key enzymatic pathways for the degradation of L-arg in mammals are *Arg1* and *Nos2*. Both are cytosolic enzymes that utilize L-arg as substrate. While *Nos2* catalyzes the formation of NO and citrulline from L-arg, *Arg1* converts L-arg into ornithine and urea. Once transported into mitochondria, the ornithine transcarbamylase (*Otc*) converts ornithine into citrulline. The ornithine aminotransferase (*Oat*), another mitochondrial enzyme, can build ornithine from glutamate and proline in the gut, whereas in other tissues, conversely, glutamate and proline are the products of this enzymatic reaction. Conversely, the combined action of the cytosolic enzymes argininosuccinate synthetase (*Ass*) and argininosuccinate lyase (*Asl*) convert citrulline again into L-arg.

Consequently, the respective tissue levels of L-arg and its metabolites are tighthly monitored. Indeed, there are several cellular components that sense and react to changes in the concentrations of amino acids such as L-arg; among these biosensors are the mechanistic target of rapamycin complex 1 (mTORC1) and some G-protein-coupled receptors (GPCRs) (Figure 1) that regulate cellular growth, metabolism, and signal integration.^3,72,73^ mTORC1 and GPCRs are both activated once L-arg is present in sufficient amounts. Vice versa, cellular stresses such as amino acid starvation activates the general control nonderepressible 2 (GCN2) stress response kinase that downregulates protein synthesis, consequently affects the phenotype of immune cells, and inversely is able to inhibit mTORC1 again.^74,75^ Thus, L-arg can also regulate cellular growth independent of the production of polyamines.

In mammals, the availability of L-arg depends on dietary uptake, catabolic degradation, regeneration of L-arg from citrulline and protein turnover. Actually, there exists a complex network of different enzymes that utilize L-arg as a substrate or synthesize it as a product.^6^ These enzymes often interact or compete with each other and can influence immune responses in multiple and even opposing ways.^5^ The key enzymatic pathways for the degradation of L-arg in mammals are the nitric oxide synthases (NOS) and the arginases (Arg) (Figure 1). Of immunologic relevance are in particular the cytosolic arginase isoform type 1 (Arg1) and the inducible isoform 2 of the three known NOS (NOS_2_). Moreover, cells may contain multiple intracellular L-arg pools that are not equally accessible to all L-arg metabolizing enzymes.^3^ Finally, the availability of L-arg is influenced by the expression, efficiency and capacity of L-arg transporters. These consist mainly of mammals of members of the solute carrier 7 family (SLC7) or other cationic amino acid transporters (CATs) (Figure 1) that transport next to L-arg a broad range of other metabolites into and out of cells.^76^

Enterocytes of the small intestine also synthesize citrulline from glutamine, proline, or ornithine (Figure 1) and thus, provide a local L-arg supply to the gut.^77^ This local L-arg supply impacts intestinal cell physiology and immune homeostasis in many ways, similarly as a dietary L-arg supplementation as discussed below. However, being a major source of endogenous L-arg synthesis, enterocytes are also pivotal for the maintenance of L-arg homeostasis in other tissues of the body and for inter- and intra-organ L-arg exchange.^12^ Consequently, supplementation of L-arg and/or inhibition of L-arg consumption can be a key issue in situations in which the gut is compromized.^12,78^

Indeed, intestinal microbiota and the hypoxic microenvironment of the gut trigger a constitutive expression of L-arg converting enzymes such as Arg1 in intestinal tissues.^52,79^ Importantly, during inflammation or infection the expression and/or function of multiple L-arg converting and/or depriving enzymes can be enhanced emptying L-arg supplies and turning L-arg into a nutritionally essential amino acid. Consequently, patients that suffer from catabolic stress might profit from dietary L-arg supplementation, as studied intensively in the past.^80^ While these studies focused on the host´s metabolism, less attention had been paid to the effects of L-arg supply on the intestinal microbiota or gastrointestinal pathogens and their consequences for metabolic interactions with the host.

## Microbial L-arg metabolism

Similar to in mammals, L-arg plays a pivotal role in multiple biological processes of microbes. Thus, L-arg is a precursor of polyamine synthesis and serves as a building block of proteins. It is also pivotal for microbial growth and differentiation, and acts as a microbial energy source.^4^ Consequently, multiple anabolic and catabolic enzymatic pathways regulate L-arg availability and metabolism in microbes as well.

**Figure 2.**
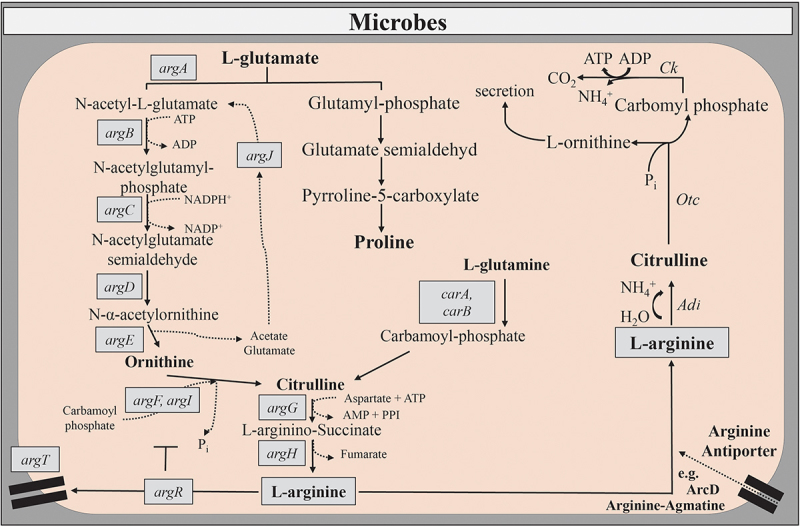
Anabolic and catabolic pathways of L-arg metabolism in microbial cells.

There exist several routes for the biosynthesis of L-arg. Two of them proceed from L-glutamate or glutamine in bacteria (Figure 2).^4^ *Enterobacteriaceae* that contain several species causing gastrointestinal diseases, use thereby the so-called linear arginine pathway in which the acetyl group removed from N-acetyl-L-ornithine (NAO) is lost to the environment during the enzymatic reaction (Figure 2). Another striking feature of bacterial L-arg biosynthesis is that L-ornithine can be formed by several non-orthologous enzymes.^81^ Moreover, arginine sensors such as ArgR (Figure 2), regulate the expression of bacterial virulence genes.^82^ Thus, L-arg is an important substrate for microbial metabolism and virulence.

L-arg can also be a source of carbon, nitrogen, and energy through a variety of catabolic pathways in bacteria, fungi, and protozoan parasites. These catabolic pathways include the arginine deiminase pathway (ADI) (Figure 2), the arginase (Arg) pathway, the arginine succinyltransferase pathway (AST) and the arginine oxidase (AO) pathway.^4^ While ADI and Arg contribute to establish infections, the role of AO and AST in microbial pathogenesis is unknown.

The ADI system (Figure 2) supplies energy and/or acid tolerance to a variety of bacteria and parasites.^83,84^ It enables *Enterobacteriaceae* to overcome oxygen limitations in the gastrointestinal tract.^85^ This arginine-dependent acid resistance system decarboxylates L-arg to L-ornithine or alternatively to agmatine, thereby consuming one proton from the cytoplasm.^86^ In protozoan parasites, ADI also acts as a virulence factor and suppresses local immune responses due to the restriction of L-arg availability.^47–49^ Next to its impact on the virulence and immune escape of gastrointestinal pathogens, ADI of probiotic bacteria can also beneficially modulate immune responses of the host and alleviate symptoms of intestinal inflammation.^87^ Importantly, microbial ADI moved also into the focus of interest because of its therapeutic potential against auxotrophic tumors.^88^

**Figure 3.**
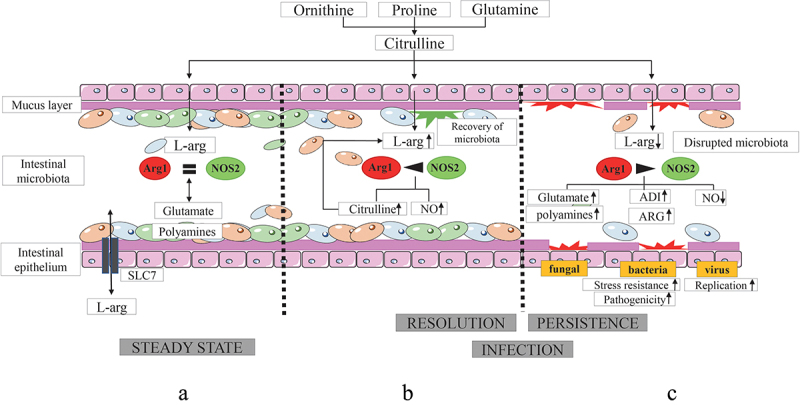
Mutual interactions of L-arg with host tissues, intestinal microbiota and pathogens and their consequences for the outcome of infection.

Arginase occurs in many bacterial genera and species. It contributes to basic nitrogen metabolism and redistribution, stress resistance and bacterial pathogenesis.^81^ Some bacteria even utilize L-arg as the sole source of carbon and nitrogen.^4^ However, not all bacteria such as *Escherichia coli* (*E. coli*) express the arginase pathway.^45^ In contrast, other bacteria belonging to the *Burkholderia cepacia* complex that encompass opportunistic human pathogens encode even more than one arginase gene.^81^ Furthermore, pathogenic bacteria including *Helicobacter pylori* strongly induce the arginase pathway to evade the immune response of the host. The resulting depletion of L-arg lowers the substrate availability for anti-microbial acting enzymes of the host (Figure 3), such as NO synthases and is thus, beneficial for bacterial survival following invasion of host tissues.^50^

In fungi, the arginase enzyme represents a major catabolic path for L-arg.^89^ With a few exceptions, most fungi do not express an arginine decarboxylase (ADC). Instead, the majority of fungi produce the polyamine putrescine via decarboxylation of ornithine, a product of the arginase reaction.^90^ Thus, fungi possess arginase-dependent and arginase-independent routes of polyamine production that do not require the production of agmatine.^1^ Importantly, the intraluminal polyamine pool is filled by collective biosynthetic pathways of different intestinal microbiota.^91^ Next to their usual functions in mammalian and microbial cell growth and differentiation, polyamines suppress specifically chronic inflammatory processes in the gut and contribute to the maintenance of intestinal barrier function (Figure 3).^92^

## Role of L-arg metabolism in microbial pathogenesis and immune evasion in the gut

As outlined above, L-arg and its products are central for multiple biological processes in mammals and microbes and play a key role in host–microbe interactions.^1–6^ However, while L-arg metabolism modulates also immune responses and mucosal barrier functions in mammals,^34^ L-arg is crucial for the growth, development, virulence, and/or (sexual) reproduction of bacteria, fungi, and parasites and is a key metabolite for viral replication.^1–6,93^ The competition for L-arg between mammalian and microbial cell needs to be therefore tightly controlled in order to maintain homeostatic host–microbe interactions, particularly at interfaces, where many microbes mutually interact with the host. Thus, an availability of L-arg in sufficient amounts balances the equilibrium between metabolic demands of microbial and host cells in the gut (Figure 3a). While sufficient amounts of intraluminal L-arg foster also the recovery of intestinal microbiota and the resolution of inflammation or infection (Figure 3b), L-arg deprivation prolongs bacterial persistence and perpetuates chronic inflammation (Figure 3c). By sensing environmental L-arg concentrations, microbes can adapt their metabolism from steady state conditions to various different stress situations. Moreover, pathogens can deplete L-arg from the environment thereby restricting substrate access for host enzymes converting L-arg into anti-microbial compounds. Finally, L-arg deprivation can disrupt intestinal microbiota and consequently impair colonization resistance allowing the outgrowth of pathogenic and pathobiontic microbial organisms (Figure 3c). Thus, disruptions of L-arg metabolism have been associated with a variety of disorders affecting intestinal permeability and the outgrowth of pathogenic/pathobiontic microorganisms, including IBD, NEC, severe infections, or sepsis (Table 1).^13,26−28,43−56,68−71^ However, L-arg is not only an integral component of the host defense and a crucial modulator of immune responses, but also directly affects bacterial virulence and pathogenesis.^94^ Not only free L-arg, but also arginine-residues in proteins play fundamental roles in these latter processes. Indeed, arginine residues modify many biological functions of proteins. Thus, we will discuss here selected examples of bacteria, parasites, viruses, and fungi that can cause intestinal infections and exploit L-arg metabolism for immune evasion or as virulence and/or survival factor.

## Bacterial pathogens

**Figure 4.**
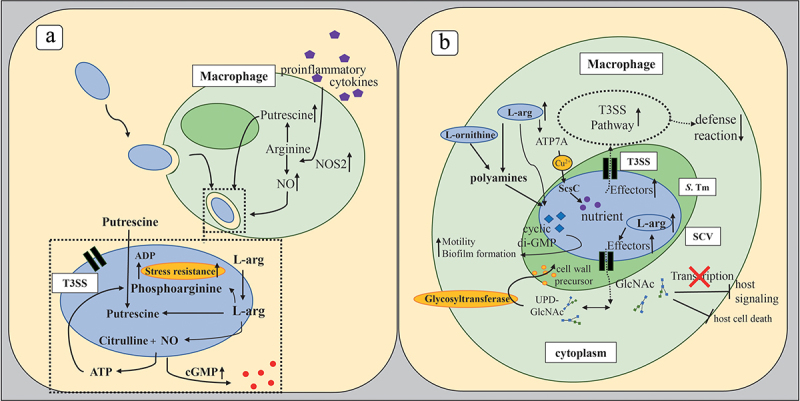
Effects of L-arg on microbial pathogenesis.

Bacterial secretion systems, particularly the syringe-like type III secretion systems (T3SSs) are fundamental tools for the interaction between some Gram negative bacteria and their hosts (Figure 4a,b). T3SSs span the inner and outer bacterial membranes and form injection nanomachines that deliver different effector proteins into target cells of their hosts in order to modulate their functions and to establish colonization and/or disease.^95,96^ Many of these bacterial effector proteins have arginine residues and share catalytic activities that promote post-translational modifications of host proteins. One family of effector proteins, for example, exhibits glycosyltransferase activity that catalyzes the addition of N-acetyl-d-glucosamine to specific arginine residues in target proteins, leading to a reduced NF-κB pathway activation and impaired host cell death and thus, providing a niche for intracellular bacteria to survive.^97^ Although L-arg metabolism interferes with many bacterial pathogens, we will focus here on three gastrointestinal pathogens, namely, *Clostridioides difficile* (*C. difficile*), *Citrobacter rodentium* (*C. rodentium*) and *Salmonella enterica* serovar Typhimurium (*S*. Tm).

- *difficile* is leading cause of antibiotic-associated diarrhea worldwide.^98^ This anaerobic and spore-forming bacterium produces two toxins, toxin A (TcdA) and toxin B (TcdB) that mediate disease. There is also epidemiologic evidence that *C. difficile* infections (CDI) occur more frequently and are more severe in IBD patients compared to the general population.^99–101^ Furthermore, asymptomatic colonization with *C. difficile* is also more frequent in the IBD population.^102^ Interestingly, L-arg metabolism is involved in the non-inflammatory colonization of intestinal surfaces with *C. difficile* as well as in inflammatory CDI. For example, mice humanized with stool samples from patients with diarrhea that still contained a healthy-like microbiota composition were recalcitrant to CDI and inflammation compared to mice humanized with feces from dysbiotic donors.^103^ Utilizing gut microbiome metatranscriptomic analysis, mice recalcitrant to CDI and inflammation exhibited an increased community-wide expression of L-arg, ornithine, and polyamine metabolic pathways.^104^ Thus, the increased availability of L-arg and its inter-related metabolic pathways enhance gut barrier function, lower the inflammatory responses of immune cells and thus, contribute indirectly to asymptomatic carriage.^14^ Furthermore, L-arg might directly inhibit the production of the two-disease mediating toxins.^105^ Vice versa, Enterococci provides fermentable amino acids, including leucine and ornithine, which increase the fitness of *C. difficile* in the antibiotic-perturbed gut. The parallel depletion of L-arg by enterococci through arginine catabolism provides thereby a metabolic cue for *C. difficile* that facilitates increased virulence.^106^ Interestingly, IBD patients suffering from concomitant CDI revealed a more pronounced dysbiosis with higher levels of *Enterococcus* operational taxonomic units (OTUs).^107^
- *C.. rodentium* is a murine pathogen utilized as a surrogate animal model to study enteropathogenic *E. coli* (EPEC) and enterohemorrhagic *E. coli* (EHEC) infections *in vivo*.^108^ *C. rodentium* and both *E. coli* pathotypes share respective pathogenic mechanisms. This also includes the expression of genes encoding the T3SS upon sensing fluctuations of L-arg concentrations (Figure 4a).^109^ Moreover, *C. rodentium* might share the enzymatic pathways of *E. coli* that allow the production phosphoarginine, which functions as a short-term energy buffer in the recovery from and adaptation to pH-induced stress (Figure 4a).^110^ At the peak of *C. rodentium* infection, for example, an increased concentration of L-arg in the colon is correlated with a downregulation of the inducible SLC7A2 transporter of the host that transports cationic amino acids such as L-arg, lysine and ornithine into host cells and is thus, a critical regulator of inflammatory processes in the gastrointestinal tract.^111–113^ This increase in intraluminal L-arg concentrations promotes the expression of virulence genes in *C. rodentium*.^82^ Interestingly, the source of L-arg sensed by *C. rodentium* was rather endogenous than dietary. In contrast, another study reported that Slc7a2^−/−^ mice exhibited an improved survival, a reduced adherence of *C. rodentium* to the intestinal lining and a decreased histopathologic injury of the gut. An inhibition of the L-arg uptake with a competitive inhibitor also prevented the attachment of *C. rodentium* to intestinal cells and the expression of chemokines.^114^ Consequently, the authors concluded that *C. rodentium* enhances its own pathogenicity by inducing the expression of SLC7A2 to favor its attachment to the epithelium and to create its ecological niche. A notable difference underlying the discrepant results of these studies is that one investigated the role of L-arg in *C. rodentium* pathogenesis at the peak of infection in a very susceptible host, while the other one assessed these parameters at the onset of disease resolution, in a less susceptible host. Thus, the genetic background, the accessibility of individual cells and of cellular compartments to L-arg as well as the intraluminal availability of L-arg for intestinal microbiota might contribute to an altered outcome in different animal models.

Bacterial transport systems involving arginine residues also influence the virulence and fitness of *C. rodentium*. For example, the twin-arginine translocation (Tat) system, that transports folded proteins across energy-transducing cytoplasmic membranes, promotes the colonization of the gut by *C. rodentium*.^115^ Substitution of both arginines in the nearly invariant twin-arginine dipeptide blocks the export of physiologic Tat substrate proteins and thus impairs microbial pathogenesis.^116^ Tat-exported peptidoglycan amidase-dependent cell division also contributed to the fitness of *S*. Tm in the inflamed gut.^117^

*S*. Tm has emerged as a model pathogen that manipulates host cells in a complex fashion. In humans, *S*. Tm causes acute intestinal inflammation similar to that in mice following pretreatment with streptomycin.^118^ Intriguingly, T3SS secreted virulence proteins also exhibit a central role in *S*. Tm pathogenesis and the induction of acute colitis.^119^ *S*. Tm encodes two specific T3SSs within the *Salmonella* pathogenicity islands 1 and 2 (SPI-1 and SPI-2) that display various important functions during host invasion and infection (Figure 4b).

Post-translational modifications of arginine residues on bacterial T3SS effectors and/or host proteins play an important role in both, physiology and pathogenesis of *S*. Tm. Similar as many other *Enterobacteriaceae*, *S*. Tm also use arginine residues for the regulation of lipopolysaccharide (LPS) biogenesis.^120^ Glycosylation of arginine residues is another characteristic feature of *S*. Tm to modify proteins post-translationally. T3SS effectors of *S*. Tm glycosylate proteins of the host and of the bacterium itself during infection of specific arginine residues with N-acetyl glucosamine (GlcNAc).^121,122^ Subsequently, the DNA binding capacity of these proteins is affected and host signaling disrupted.^123^ Furthermore, bacterial targets are modified. The functional consequences of these glycosylation processes have remained largely unclear. However, arginine-glycosylates might antagonize apoptosis-induced host cell death or might contribute to the maintenance of sufficient levels of uridine diphosphate N-acetylglucosamine (UDP-GlcNAc) which is required by glycosyltransferases as coenzyme for the synthesis of bacterial cell wall precursors.^124–126^

Similar as *C. rodentium*, *S*. Tm also senses periplasmic, free L-arg. L-arg induces a rapid and translation-independent increase of cyclic-di-GMP in *S*. Tm (Figure 4b), a second messenger, which regulates bacterial motility and biofilm formation.^127^ Furthermore, periplasmic L-arg regulates also the (stress) response of *S*. Tm to copper,^128^ an important micronutrient, but also toxic component when present in excess. Opposite to *C. rodentium*, L-arg does not appear to affect the expression of T3SS genes in *S*. Tm directly. However, metabolic products of L-arg such as polyamines might exhibit similar effects.^129^ Importantly, the depletion of polyamine biosynthesis led to a down-regulation of genes encoding structural components of the T3SS 1 and its secreted effectors. Thus, L-arg might be indirectly involved in the regulation of T3SS in *S*. Tm as well.

The metabolism of L-arg also contributes to the adaption of enteric pathogens such as *S*. Tm to a hostile environment. Thus, *S*. Tm can colonize the gut despite the required stomach passage with its lethal acidic pH environment (pH < 2.5) due to the activation of inducible acid tolerance response (ATR) systems. The biodegradative arginine decarboxylase (ADC) of *S*. Tm is a pivotal component of this ATR system.^130^ The enzyme consumes cytoplasmic protons in the process of L-arg degradation to agmatine.^86^ Consequently, the arginine-agmatine antiporter (AdiC) exchanges the product agmatine for L-arg again. Thus, the ADC pathway in *S*. Tm permits survival under acidic conditions as low as pH 2.5.^131^

The ADI system is another L-arg dependent pathway that contributes to the virulence of *S*. Tm (Figure 1). The expression of ADI pathway genes is induced inside macrophages. Enzymes of the ADI pathway promote next to their metabolic functions the intracellular replication of *S*. Tm. and the virulence of this gastrointestinal pathogen during infection of mice.^132^ However, the deletion of genes in the ADI pathway did not affect the production of NO by macrophages.

## Parasitic pathogens

There are also several parasites, which primarily infect the intestinal tract. These include a variety of protozoan parasites and a panoply of helminths. We will focus here exemplarily on two protozoan parasites, namely *Giardia* (*G*.) *lamblia* and *Cryptosporidium* (*C*.) *parvum* and discuss the role of the L-arg metabolism on their pathogenesis. Since L-arg exhibits a myriad of biological roles, we will also discuss its role as a substrate of different enzymatic reactions.

The protozoan parasite, *G. lamblia* is the causative agent of giardiasis, a frequent cause of diarrhea worldwide. It primarily interacts with the local microbiota, the mucus and the epithelial cell layer in the gut.^133^ Indeed, the composition of the intestinal microbiota is known to affect the outcome of *G. lamblia* infection.^134^ Vice versa, *G. lamblia* infection also alters the microbial diversity of the gut microbiota.^135^

Although an infection usually induces a minor inflammatory response, giardiasis is associated with enterocyte apoptosis, hypermobility, intestinal barrier dysfunction, and villus shortening.^136–138^ However, in genetically susceptible hosts even a transient infection with this pathogen can trigger colitis.^139^ Despite extensive *in vitro* investigations, the dynamics of infection between the host and the parasite *in vivo* underlying acute or chronic giardiasis are only poorly understood. A metabolite that might influence the clinical outcome is L-arg, as it can simultaneously affect the intestinal microbiota, the parasite itself as well as the immune response of the host.

Indeed, *G. lamblia* can affect L-arg metabolism in many ways. First, it is able to degrade L-arg directly as energy source via the arginine dihydrolase pathway.^84^ *G. lamblia* release also two enzymes of this pathway (Figure 3), the arginine deiminase (ADI) and the ornithine carbamoyltransferase (OCT) following interactions with intestinal epithelial cells (IECs).^140^ The resulting diminished availability of L-arg for the host reduces the proliferation of IECs and of T lymphocytes.^47,48^ Second, this protozoan parasite also indirectly influences the availability of L-arg due to the induction of host enzymes utilizing L-arg as substrate. Thus, Arg1- and NOS2-expressing macrophages accumulate in the gut following *G. lamblia* infection.^141^ As both enzymes compete for L-arg as common substrate, Arg1 expression could limit the availability of L-arg for NOS2 and thus promote the growth of *G. lamblia*, since NO acts cytostatic on the trophozoites of *G. lamblia in vitro* and limits encystation and excystation *in vivo*.^142^ Therefore, a reduction of the NO response favors the growth of *G. lamblia* and the parasite could utilize the induction of Arg1 as immune evasion strategy. Indeed, an addition of L-arg or of citrulline that converts into L-arg once having entered intestinal tissues reverses many of the adverse effects of L-arg depletion. Thus, dietary L-arg substitution could be a beneficial supplement in oral rehydration therapy of giardiasis.

*C. parvum* is the leading cause of human cryptosporidiosis, which can manifest with intestinal or respiratory tract symptoms such as diarrhea or cough.^143^ Similar as *G. lamblia*, *C. parvum* can infect humans and a wide range of animals.^143,144^ Furthermore, the gut microbiota and/or intraluminal metabolites affect the outcome of *C. parvum* infection as well.^145–148^ Vice versa, commensal strains of *Cryptosporidia* such as *Cryptosporidium tyzzeri* protect against *C. rodentium* infection due to a modulation of intestinal immune responses.^149^ Thus, multiple microbial genera and pathways interfere in multiple ways with each other.

The protective effects of L-arg metabolism on the clearance of *C. parvum* have been primarily associated with the NOS isoforms.^150–153^ In contrast, the expression of Arg1 is correlated with an enhanced inflammation of the gut following cryptosporidial infection.^154^ Vice versa, the expression of Arg2 by the intestinal epithelium promoted the uptake of L-arg from the intestinal lumen following *C. parvum* infection, which might initiate the synthesis of polyamines that are required for the repair of damaged mucosal tissues.^155^

Other indirect effects of L-arg were linked to diamine oxidase (DAO), a polyamine-degrading enzyme whose expression positively correlates with the growth of mucosal cells in the small intestine.^156^ Low serum or plasma activities of DAO predict intestinal mucosal damage in human and veterinary medicine.^157–159^ Interestingly, plasma concentrations of L-arg as precursor substrate of polyamines are also significantly decreased in diarrheic calves compared to controls.^160^ Thus, these data indicate a dysfunction of L-arg metabolism upon *C. parvum* infection and subsequently suggest L-arg and its metabolites as biomarkers for mucosal damage in humans or animals suffering from cryptosporidiosis.

## Viral pathogens

The role of L-arg on viral replication is controversely discussed in the literature. On the one hand, L-arg can inactivate some viruses due to interactions with their lipid membranes.^161,162^ Moreover, L-arg produced by intestinal microbiota can enhance mucosal γδ T cell responses to viral infections.^163^ On the other hand, being a key metabolite for viral replication, L-arg depletion has been suggested as potent antiviral strategy against several viruses (summarized in^93^. Unfortunately, only limited data on the effects of L-arg on intestinal viral infections or on the intestinal viriome are available that has been associated with IBD pathogenesis.^164–166^

However, several studies addressed the role of L-arg metabolism in the pandemic COVID-19 disease, caused by SARS-CoV2. Next to respiratory disease, this novel beta coronvirus can also cause gastrointestinal symptoms.^167,168^ Indeed, angiotensin-converting enzyme 2 (ACE2) the receptor through which SARS-CoV2 enters the host cells^169,170^ is not only found in the airways, but also on enterocytes.^171,172^^173–182^ Following infection, SARS-CoV2 significantly alteres the metabolism of L-arg. For example, COVID-19 patients had significantly lower plasma L-Arg concentrations compared to healthy controls.^60^ Vice versa, methylated arginine products such as ADMA, SDMA, or n-monomethyl-1-arginine (L-NMMA) were significantly higher in patients with severe Covid-19.^62^ Importantly, the variations in COVID-19 severity and the fluctuating involvement of different organ systems might be a result of phenotypic variations in L-arg metabolism to which heritable metabolic pathways in erythrocytes contribute.^63^ Indeed, adding oral L-arg to standard therapy can decrease the length of hospitalization and reduce respiratory support in defined patient cohorts (Table 2).^183,184^ Furthermore, L-arg supplementation contributes to the reduction of oxidative stress and of endothelial dysfunction in long-Covid-19 sequelae.^185^

**Table 2.** Clinical trials assessing the effects of L-arg supplementation on selected infectious agents, sepsis, IBD and NEC.

Disease	Study design	Adminis-tration	Concen-tration	Dura-tion	Clinical effect	Ref
Sepsis	n = 390 prospective, randomized double-blinded clinical trial	within 72 hours after intensive care unit (ICU) admission enteral nutrition	1,25 g L-arg/100 ml (total volume 2.5 L/day) → 31,25 g/day	until ICU discharge	reduced morbidity rate, no effect on mortality in critically ill patients	^173^
	n = 11 prospective clinical study	infusion of the NO synthesis inhibitor NG-nitro-L-arginine methyl ester (L-name)	1 mg L-name/kg/h	12 hours	increased systemic vascular resistance; no impact on inflammatory cytokine or NO	^174^
NEC	n = 237 randomized multicentre clinical trial	enteral nutrition	6.8 g/L	28 days	reduced mortality	^175,176^
	n = 152 randomized placebo-controlled study	oral	1.5 mmol/kg per day as parenteral nutrition	28 days	reduced incidence of all stages of NEC in premature infants	^177^
	n = 285 Cochrane Neonatal Review → summary of (quasi-) randomized controlled trials	oral or enteral	1.5 mmol/kg/d, 0.75 mmol/kg/d	at least 7 days	reduced risk for development of NEC; reduced death rate related to NEC	^178^
Bacterial infection review of evidence	n = 912 (summary of 8 studies)	enteral and/or oral nutrition pre-operative	n.d.	5–7 days before surgery	reduction of postoperative infections, reduced length of hospital stay	^64^
→ summary of rando-mized trials	n = 1483 (summary of 13 studies)	enteral and/or oral nutrition peri-operative	3–12,5 g	from 10 days before until 14 days after surgery	reduction of postoperative infections, reduced length of hospital stay	^64^
	n = 1483 (summary of 17 studies)	enteral and/or oral nutrition post-operative	1–25 g	1–11 days after surgery	reduction of postoperative infections, reduced length of hospital stay	^64^
Fungal infection	n = 120 randomized clinical trial	nutritional support intervention	8.5 g/L containing enteral nutrient solution	14days	alleviation of malnutrition promotion of immune functions and wound healing recovery of intestinal motility and barrier function	^179^
IBD	CD: *n* = 31 randomized controlled trial	nutritional isocaloric supplements	3.8 g/2× per day	9 weeks	improved nutritional status	^180^
Covid-19	n = 80 parallel randomized study	oral	3 g/day	21–30 days	reduction in ventilation days decreased muscle reduction	^181^
	n = 1390 nationwide survey	oral	1,6 g/twice per day	30 days	reduction of long-Covid symptoms	^185^
	n = 74 randomized controlled trial	oral	3 g/day	max. 10 days	no signifcant advantages	^184^
	n = 50 randomized controlled trial	oral	1,6 g/twice per day	28 days	improved physical performance, endothelial function and less persistent fatigue in Long Covid	^182^

However, no data are available on the role of L-arg metabolism and/or supplementation on Covid-19-mediated intestinal disease. Nonetheless, intraluminal L-arg metabolism and microbiota might affect obesity or hamper the intestinal barrier and thus promote the subsequent formation of neutrophil extracellular traps (NETs) due to the translocation of microbial products,^186–188^ with obesity and NETs being risk factors for severe COVID-19 disease.^189–193^ Furthermore, as L-arg modulates intestinal microbiota^14,24,52,194,195^ and as microbiota can affect immune responses in the lung, as discussed below also for bacterial and fungal infections,^196–199^ dietary L-arg supplementation and subsequent alterations in intestinal microbiota composition/function might indirectly improve the outcome of pulmonary SARS-CoV2 infection.

Moreover, SARS-CoV-2-induced endothelial dysfunction leads to endotheliitis and thrombosis due to endothelial nitric oxide (NO) inhibition with subsequent vasoconstriction and tissue hypoxia. Thus, being a precursor of NO L-arg could improve endothelial function due to the prevention of SARS-CoV2 induced coagulopathy and/or the NO-mediated inhibition of viral replication.^200^ In contrast, as L-arg is a key metabolite in the life cycle of many viruses, L-arg depletion might be also a promising therapeutic approach for patients with Covid-19.^93^

In summary, L-arg interferes with various aspects of viral infection and virus associated complications, including viral replication, virus-triggered immune responses, coagulation, and endotheliitis. Thus, viruses can simultaneously affect compartmentalized and systemic L-arg metabolism.

## Fungal pathogens

Fungi are an integral part of the commensal gut microbiota in humans and mice.^201–205^ Similarly to bacteria, environmental, and mother-to-infant transfers of fungi occur perinatally, resulting in the rapid colonization of different body sites.^203^ There, the mycobiota influences the assembly and function of the commensal microbiota and the development of the immune system.^203,206,207^ Later on, the diet primarily influences the colonization of the intestinal tract by fungi and thus, the composition of the mycobiome.^205,207^ Presence of *Candida spp*., for example, is positively associated with dietary carbohydrates and correlates inversely with dietary amino acids, proteins, and fatty acids.^205^ Thus, the gut mycobiome appears less stable than the bacterial microbiome, and is even more likely subject to environmental factors.^204^ Interestingly, fungal and bacterial abundances in the gut appear to negatively correlate to each other.^208^

Similarly, as described for the outgrowth of *C. difficile* ^209^ a disruption of the physiologic commensal microbiota is also a prerequisite for the overgrowth of fungi in the gut.^207,208^ Importantly, dysbiosis also affects intestinal fungi is associated with invasive infections and IBD.^205,210^ Thus, most fungi are intestinal commensals and contribute to the maintenance of mucosal immune homeostasis and colonization resistance. Using the common mucosal surface commensal *Candida* (*C*.) *albicans* as an example, we will discuss here briefly some aspects of this double-edged function of fungi in health and disease.

For example, *C. albicans* is a potent inducer of Th17 responses that can exhibit beneficial functions in the gut and can contribute to colonization resistance.^211^ Indeed, the commensal *C. albicans* pool generates protective immunity against a wide range of opportunistic fungal and obligate bacterial pathogens.^212–214^ Vice versa, a *C. albicans* mediated, systemic expansion of Th17 cells that cross-react to antigens of other fungi, such as *Aspergillus fumigatus* or to antigens of house dust mite can cause immunopathology of the lungs.^198,199^ Moreover, *C. albicans* might contribute to the accumulation of Th17 cells in intestinal tissues of IBD patients. Indeed, *Candida* is the most prevalent fungal genus consistently increased in several IBD cohorts based on fecal sequencing.^215–218^

All *Candida spp*. can employ amino acid metabolism. However, there exist metabolic differences between individual *Candida spp*.^219^ L-arg, for example, is crucial for fungal growth, development, pathogenicity and survival.^1^ Moreover, phagocytosed *C. albicans* rapidly upregulate the biosynthesis of L-arg upon exposure to reactive oxygen species.^220^ The subsequent increased availability of L-arg resulted in a switch from yeast to hyphae and enabled *C. albicans* to escape macrophage-mediated host defense.^221^ In addition, *C. albicans* induces the expression of Arg1 in human mancrophages and thus, limits the availability of L-arg for NOS2. This resulted in a reduced NO release and subsequent improved fungal survival.^222^ Vice versa, L-arg inhibits the formation of biofilms in fungi.^223^ Furthermore, dietary L-arg substitution might not only exhibit anti-colitogenic effects, but also limit the adverse effects of systemic Th17 responses that are inversely correlated with the availability of L-arg.^52,57^

## Influence of L-arg on gut microbiota, mucosal immune responses, and the outcome of intestinal disease

Dietary proteins modlate the composition and the metabolic activity of the gut microbiota, which in turn influences human health. Vice versa, the microbiota also affect protein metabolism and thus, the bioavailability and distribution of free and protein-bound L-arg.^224^ These mutual interactions influence the epithelial and the endothelial barrier of the gut, affect colonization resistance, and control the translocation of intraluminal contents into the portal circulation, potentially leading to a variety of metabolic or immune-mediated disorders.^225–227^ However, the role of specific microbiota and/or microbial pathways in these complex interactions is just begun to be understood.

**Figure 5.**
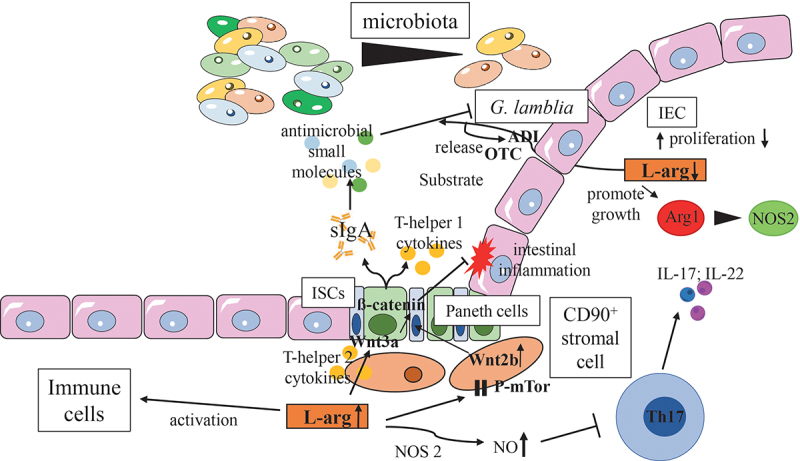
Effects of L-arg on commensal microbiota, the intestinal epithelium and mucosal immune homeostasis.

The interplay of intestinal microbiota and L-arg-metabolizing enzymes is involved in the pathogenesis of intestinal and extra-intestinal disorders, including autoimmunity, cancer, and infectious disease.^194^ Comparing stool metagenomics from rural through urban populations, a recent study connects L-arg metabolism with the enrichment of specific microbiota and potential immunomodulatory pathways.^195^ Accordingly, L-arg directly enhances the diversity and richness of intestinal microbiota in pre-clinical models and subsequently hampers the colonization of the gut by intestinal pathogens.^24,52,228,229^ Moreover, L-arg promotes mucosal immunoglobulin and cytokine production^228,229^ and influences immune cell phenotypes and functions.^194,230^ Thus, L-arg influences directly and indirectly colonization resistance mechanisms (Figure 5). Vice versa, as described above, intestinal pathogens can exploit L-arg to evade the immune response or to enhance their own pathogenicity.^50–132^ Moreover, L-arg depletion disrupts intestinal microbiota and thus, mucosal colonization resistance (Figure 3c). Thus, L-arg can simultaneously influence immune responses of the host and microbiota-mediated colonization resistance as well as pathogen replication and virulence.

Interestingly, in this context, and in contrast to the intestinal pathogens described above, the bacterial ADI system can even serve as a therapeutic component rather than as virulence factor in the probiotic *Enterococcus faecium* strain SF68. Thus, ADI of SF68 inhibits the activation of NFkB and JNK (AP-1) pathways in intestinal epithelial cells.^87^ Consequently, ADI may contribute to the anti-inflammatory and immunomodulatory effects observed in prior clinical human and animal SF68 trials.^231,232^ Interestingly, *Saccharibacteria*, a group of widespread and genetically diverse ultrasmall bacteria with highly reduced genomes acquire the ADI system during their transition from environmental to mammalian niches.^233^ Thus, the ADI system might enable bacteria to survive in a hostile environment. Dependent on the pathogenicity of the bacterium itself the outcome of bacterial ADI activation or acquisition might be beneficial or detrimental for the host.

Moreover, L-arg derived substrates such as NO modulate the interplay of commensal microbiota and intestinal pathogens. For example, NOS_2_-derived NO and peroxynitrite have been broadly associated with anti-microbial effects.^5^ Accordingly, NOS_2_ protected from invasive *S*. Tm infection *in vivo* and *in vitro*.^234,235^ However, the anti-microbial active peroxynitrite can be quickly converted into nitrate during its diffusion into the intestinal lumen where *S*. Tm profits from nitrate respiration.^236^ Nitrate generated as a by-product of an inflammatory response also conferred a growth advantage to *E. coli*.^237^ Thus, both bacteria can even expand in the gut lumen during inflammation. *E. coli* competes thereby with *S*. Tm for nitrate derived from intestinal epithelial cells, but not for nitrate derived from phagocytic infiltrates and thus, confers colonization resistance only in a distinct intestinal niche.^238^ However, *E. coli* similar as other *Enterobacteriaceae* markedly expand in IBD patients compared to healthy controls.^236^ Indeed, NO derived from dysbiotic nitrate respiration is involved in the pathogenesis of IBD and alters colon motility.^239,240^ In contrast, the bioactivation of dietary nitrate by certain commensal microbiota might still promote favorable metabolic effects, even under inflammatory conditions.^241^ Therefore, the ability of selected bacteria to reduce nitrate certainly contributes to the changes in the microbial community structure observed during intestinal infection or chronic inflammation. These changes included the depletion of obligate anaerobic bacteria and an increase of facultative anaerobic pathobionts from the family of *Enterobacteriaceae*. Thus, in summary, L-arg and its products promote or impair colonization resistance in a context-dependent manner. Consequently, further studies need to identify the microbiota that selectively profit from L-arg supply. Moreover, the individual beneficial functions of selected microbiota against intestinal infection and inflammation as well as the microbial pathways underlying protection in the respective intestinal disorders need to be characterized.

## Effects of dietary L-arg on intestinal immune responses

Intestinal microbiota as targets for L-arg metabolism has become a focus of research within recent years as well.^242^ For example, dietary L-arg supplementation ameliorated and resolved faster a dextran sulfate sodium- (DSS-) induced colitis in pre-clinical models.^52^ Protection from severe intestinal pathology was associated with an altered composition of intestinal microbiota and an accumulation of bacterial genera associated with human health. Furthermore, endothelial cell functions improved and less inflammatory cells were extravasated into intestinal tissues following L-arg application. Thus, oral L-arg substitution is an attractive target for clinical intervention in IBD.^243^

Dietary L-arg supplementation restores also the diversity of intestinal microbiota and protected from intestinal injury and inflammation following *C. rodentium* infection.^24^ L-arg-mediated protection was associated with an enhanced abundance of *Bacteroidetes* and a decrease of *Verrucomicrobia*. Furthermore, dietary L-arg inhibited the activation of colitogenic Th17 cells and the induction of experimental colitis by *Eggerthella lenta*,^57^ a human gut actinobacterium enriched in patients with immune-mediated diseases.^244–247^ Finally, L-arg supplementation enhances secretory immunoglobulin A (sIgA) production as well as the release of cytokines and anti-microbial peptides (Figure 4).^228,229^ This effect also depends on intestinal microbiota, as the application of antibiotics abrogated the influence of L-arg supplementation on sIgA secretion. L-arg reduces bacterial loads in mouse mesenteric lymph nodes and decreases bacterial invasion into the mouse ileal wall as well. Next to *T*- and B-lymphocytes, L-arg also influences the phenotypes and functions of other immune cells including macrophages, granulocytes, myeloid-derived suppressor cells (MDSCs), natural killer (NK), innate lymphoid cells (ILCs) and dendritic cells (DCs).^194,230^ Interestingly, oral administration of L-arg and the subsequent remodeling of gut microbiota enhances the anti-microbial immune defense even in extra-intestinal tissues, such as the lung, against non-tuberculous and tuberculous mycobacterial infection.^196,197^

As L-arg is a precursor of many metabolites, it also influences the function and phenotype of non-hematopoetic cells. Consequently, L-arg contributes to the maintenance and restoration of intestinal immune homeostasis.^45^ Thus, L-arg promotes an expansion of intestinal stem cells (ISCs) (Figure 5).^248,249^ An increased expression of Wnt2b in CD90^+^ stromal cells, a ligand of the Wnt/β-catenin signaling pathway, mediated the effects of L-arg on ISCs function.^248^ Paneth cells, a critical component of the ISCs niche, also augmented ISCs function in response to L-arg.^249^ Furthermore, intraluminal available L-arg protected the gut against 5-fluorouracil- (5-FU-) mediated tissue injury, one of the most commonly used drugs to treat cancer and a well-known agent for the destruction of the small intestinal epithelium.^250^ In contrast, L-arg supplementation of fibroblasts might have adverse effects on disease outcome as it promotes fibrosis.^251^ Thus, the cellular compartments and subsets that can be accessible to L-arg need to be characterized before a dietary supplementation of this semi-essential amino acid is initiated.

## Dietary L-arg supplementation as therapeutic option for intestinal pathologies

As a supplement, L-arg can be administered intravenously, orally or topically. Due to its broad physiologic effects and its potential benefits on endothelial and vascular dysfunction, hypertension, immune cell physiology, metabolic pathways, or wound repair,^3,65^ L-arg supplementation was evaluated in a broad range of various different diseases.^9–11,64,65,252,253^ A few clinical trials also assessed the effects of L-arg in decreasing complications of IBD, NEC, infection, or sepsis (Table 2).^28,56,68–70^ However, previous studies analyzed primarily the systemic or nutritional and less the local effects of L-arg supplementation. Thus, with the exception of animal studies,^14,24,52,113^ there are hardly any data of dietary L-arg alimentation on intestinal physiology and the composition of gut microbiota in humans available. Moreover, there exists only limited information regarding the safety and efficacy of L-arg supplementation in patients with different diseases.^252,253^ Thus, further studies need to characterize the intraluminal (adverse) effects of L-arg supplementation on colonization resistance and immune modulation, particularly with respect to its potential use in the correction of intestinal dysbiosis preceding *C. difficle* infections or IBD symptoms as alimentary adjuvant.

Furthermore, the route of application and the dose of L-arg supplied are pivotal for the anti-colitogenic or the richness and diversity of microbiota promoting the effects of L-arg. As patients suffering from ulcerative colitis (UC), one of the two main clinical entities of IBD, for example, exhibit increased L-arg levels in their sera,^53^ systemic L-arg infusion might be even detrimental in this patient cohort. Furthermore, intravenous infusion does not change the composition of the intestinal microbiota, which mediates the observed beneficial effects of dietary L-arg alimentation.^24,52^ Thus, pathologic alterations in the availability of L-arg in different host tissue and/or microbiota compartments might completely disrupt the entire L-arg metabolism. Consequently, L-arg formulations and therapeutic regimens need to aim to replenish only the compartment lacking L-arg. Therefore, the dose of dietaryly supplied L-arg needs to be optimally suited to allow beneficial changes in the composition of intestinal microbiota and/or intraluminal microbial metabolites without causing systemic spillover effects into other host tissues. Preparation of a retarded and tissue-targeted L-arg release in the gut might be advantageous in this context. In contrast, to overcome the general L-arg – deficiency as observed during sepsis or NEC, for example, a systemic reconstitution of L-arg appears to be preferable that can also ameliorate endothelial dysfunction and severe catabolism.^26–28,69^ Thus, L-arg needs to be prepared, dosed, and applied in a way that only the appropriate, L-arg deficient compartment(s) are fed.

Furthermore, alterations in the composition of microbiota can affect many other disorders including colorectal carcinogenesis.^254^ Thus, for example, an upregulation of the expression of the argininosuccinate lyase (ASL), the only enzyme able to produce Larg, correlated with an improved epithelial integrity and alleviation of colitis of inflammation-associated colon cancer.^255,256^ Epithelial argininosuccinate synthetase, in contrast, was dispensable for intestinal regeneration and tumorigenesis.^257^ Therefore, additional studies on the effects of Larg expanded microbiota or Larg mediated alterations in microbiota functions on other intestinal and extra-intestinal diseases have to be carried out.

Importantly, several bacterial, fungal, and parasitic pathogens and pathobionts also sense L-arg levels and might expand following L-arg supplementation and thus, adversely affect the outcome of disease. For example, the exclusive colonization of mice with commensal *C. albicans* reduces the susceptibility to DSS colitis or subsequent *C. difficile* infection.^206,258^ Vice versa, bacterial and fungal co-colonization or oral application of *C. albicans* into B6 mice kept under SPF conditions increased colonic inflammation.^206,259^ Moreover, *C. albicans* induced mucosal dysbiosis can also promote invasive infections.^260^ Thus, fungi influence microbial ecology in the gut in a context-dependent manner and can influence inflammation and colonization resistance in opposing ways. Consequently, explicit attention has to be paid to fungi when translating pre-clinical studies into human disease, since the diverse microbiota of free-living mammals significantly differ from the restricted microbiota of lab animals.^85,261^ In particular, fungi have been shown to promote a more human-like immunity and physiology, microbiome assembly, and interkingdom interactions between bacterial and fungal communities, affecting also the exchange of amino acids.^206–264^ Thus, the microbial composition of the gut, the niches in which microbes reside in as well as the accessible cellular compartments of the host and metabolic pathways need to be characterized. As L-arg is an important modulator of microbial virulence, we suggest to screen intestinal microbiota for the presence of pathogenic microbial organisms and pathobionts before dietary L-arg supplementation is initiated. Moreover, pre-clinical and clinical studies need to address also the role of L-arg metabolism on the intestinal mycobiome and viriome and its implications in the pathogenesis of inflammatory and infectious diseases.

## Conclusion and future directions

There exist multiple lines of evidence that mammalian and microbial L-arg metabolisms interferes with each other. While the influence of L-arg on the immune response of the host and on the pathogenesis of gastrointestinal pathogens is relatively well established, little to nothing is known about the effects of L-arg on beneficial commensal microbiota and colonization resistance in the gut. Specifically, it will be interesting to study which pathways specific microbiota engage and how microbial diversity increases following dietary L-arg supplementation. Furthermore, future studies need to evaluate whether and to which extent an altered microbiota composition and/or metabolism affects intestinal, extra-intestinal and systemic symptoms of gastrointestinal pathogens, NEC or IBD. Moreover, only limited information is available for parasitic infections that profit from L-arg supplementation in pre-clinical models.^265^ In this context, it will be important to understand how alterations in the microbiota enhance intestinal and extra-intestinal immune responses and maintain mucosal and systemic immune homeostases. Thus, L-arg supplementation might be beneficial for the outcome of many infectious and/or inflammatory diseases. Moreover, as intestinal dysbiosis and the leaky gut syndrome accompany many immune-mediated disorders beyond IBD,^21^ L-arg-mediated correction of intestinal permeability and/or microbiota composition and function might be beneficial in a variety of other immune-mediated disorders as well and could be considered as adjuvant alimentary therapy.

Cationic amino acid transport systems of the SLC7 or CAT families transport L-arg across intestinal cellular membranes in mammals. Both transporter systems can interfere with signaling in L-arg biosensor systems. Cellular components that sense and react to changes in the concentrations of L-arg are the mechanistic target of rapamycin complex 1 (mTORC1) and some G-protein-coupled receptors (GPCRs). mTORC1 and GPCRs are both activated once L-arg is present in sufficient amounts. Vice versa, cellular stress such as amino acid starvation activates the general control nonderepressible 2 (GCN2) stress response kinase that downregulates protein synthesis, consequently affects the phenotype of immune cells, and inversely inhibits mTORC1.

Abbreviations: ATP, adenosine triphosphate; CAT, cationic amino acid transporter; CO2, carbon dioxide; *Cps*, Carbamoyl phosphate synthetase; NH_3_, ammonia; NO, nitric oxide; *Nos2*, inducible nitric oxide synthase; *Oat*, ornithine aminotransferase; ODC, ornithine decarboxylase; *Otc*, ornithine transcarbamylase; *Prodh*, Proline dehydrogenase; *P*-5-C, Pyrroline-5-carboxylate; *P5cdh*, Pyrroline-5-carboxylate dehydrogenase; *P5cr*, Pyrroline-5-carboxylate reductase 1; *P5cs*, Pyrroline-5-carboxylate synthase; SLC7, solute carrier 7

A major pathway of arginine biosynthesis consists of a multi-enzymatic process network that the acetylation of the amino group of glutamate initiates. Shown here are the most pivotal steps of this so-called linear arginine pathway that is in detail described elsewhere.^266^ Conversely, a major catabolic pathways in microbial cells is the arginine deiminase pathway (ADI). The ADI system is an arginine-dependent acid resistance system to foster microbial pathogenesis and virulence. Furthermore, ADI generates cellular energy in different bacteria and parasites. Three enzymes, arginine deiminase (*Adi*), carbamate kinase (*Ck*), and ornithine transcarbamylase (*Otc*) constitute the ADI system. The respective enzymatic reactions catalyze the conversion of L-arg into ornithine, ammonia, and carbon dioxide along with the concomitant formation of ATP. The arginine-ornithine antiporter (ArcD), as part of the ADI, mediates the uptake of L-arg and the concomitant export of ornithine in an ATP-independent manner. To acquire arginine from the host intestinal pathogens such as *S*. Tm exploit also the arginine-agmatine antiporter or the lysine-arginine-ornithine-binding periplasmic protein ArgT. The transcriptional regulator and arginine sensor ArgR controls arginine metabolism and transport, for example, due to the regulation of ArgT expression or the activation of catabolic pathways.

Abbreviations: *Adi*, arginine deiminase; ADP, adenosine diphosphate; ArcD, arginine-ornithine antiporter; *argA*, N-acetylglutamate synthase; *argB*, N-acetylglutamate kinase; *argC*, N-acetyl-γ-glutamyl-phosphate reductase; *argD*, aceylornithine aminotransferase; *argE*, acetylornithine deacetylase; *argF*, *argI*, ornithine transcarbamylase; *argG*, argininosuccinate synthetase; *argH*, argininosuccinate lyase; *argJ*, ornithine acetyltransferase; *argR*, arginine deiminase system associated receptor; ATP, adenosine triphosphate; *CarA*, *CarB*, carbamoyl phosphate synthase; CO_2_, carbon dioxide; *Ck*, carbamate kinase; NH_4_, ammonium

Bacterial, and mammalian enzymes compete for L-arg as common substrate and convert it into various metabolites that exhibit diverse and even opposing effects on the immune response, colonization resistance or microbial virulence. Both enterocytes and microbiota can be a cellular source of local L-arg in the gut. While host and microbial enzymes balance L-arg metabolism under steady state conditions (A), infection/inflammation disrupts this homeostasis resulting in an enhanced L-arg consumption and/or decreased L-arg production (C). While sufficient amounts of L-arg foster the resolution of inflammation/infection and the recovery of microbiota (B), L-arg deprivation prolongs bacterial persistence and/or perpetuates chronic inflammation (C). Microbial pathogens frequently utilize L-arg to induce their ADI system, which helps them to survive within hostile environments. Furthermore, microbial pathogens exploit the arginase system to evade the immune response of the host. The subsequent depletion of L-arg from the environment hampers the immune defense of the host and promotes the survival of bacteria and parasites inside host cells in addition. Furthermore, L-arg deprivation disrupts the functions and diversity of intestinal microbiota subsequently hampering colonization resistance.

Abbreviations: ADI, arginine deiminase; ARG, microbial arginase; Arg1, arginase isoform 1; NO, nitric oxide; NOS2, inducible nitric oxide synthase; SLC7, solute carrier 7

Arginine can affect microbial pathogenesis and the anti-microbial immune defense in multiple ways.

- Arginine can be a source of polyamines, such as putrescine that promote microbial growth. Moreover, L-arg derived phosphoarginine functions as a short-term energy buffer in the recovery from and adaptation to pH-induced stress. Vice versa, L-arg can be a source for anti-microbial NO, generated by host NOS2.
- Arginine residues of bacteria or host cell-derived proteins are targets for modification by glycosylation. These modifications affect either the functions of effector proteins transported through bacterial T3SSs or the biology of host proteins. Thus, proteins from both sources can be glycosylated with consequent diverse biologic effects, which include the inhibition of host cell death or the promotion of intracellular bacterial survival. Glycosylation might also maintain sufficient levels of uridine diphosphate N-acetylglucosamine (UDP-GlcNAc) which is required by glycosyltransferases as coenzyme for the synthesis of bacterial cell wall precursors. Bacterial motility and biofilm formation increase due to the accumulation of secondary bacterial messengers such as cyclic di-GMP. Importantly, many pathogenic bacteria sense L-arg concentrations and consequently induce the expression of T3SS genes or other virulence factors that disrupt the immune defense of the host.

Abbreviations: ADP, adenosine diphosphate; ATP, adenosine triphosphate; ATP7A, copper transporting ATPase; Cu^2+^, copper ions; di-GMP, dimeric guanosine monophosphate;GlcNAc, N-acetylglucosamine; NO, nitric oxide; NOS_2_, inducible nitric oxide synthase; ScsC, periplasmic suppressor protein of copper sensitivity; SCV, *Salmonella* containing vacuole; *S*. Tm, *Salmonella* Typhimurium; T3SS, type 3 secretion system; UDP-GlcNAc, uridine diphosphate N-acetylglucosamine

The local endogenous L-arg supply by enterocytes as well as dietary L-arg supplementation exhibit various beneficial effects on intestinal physiology and immune homeostasis. First, both enhance the richness and diversity of intestinal microbiota. Second, both promote the release of sIgA, anti-microbial peptides and cytokines that reciprocally shape the composition of intestinal microbiota. Third, both inhibit the expansion and differentiation of colitogenic Th17 cells, at least partially due to the induction of NOS_2_. Finally, L-arg promotes the expansion and renewal of intestinal stem cells (ISCs). Paneth cells and Wnt2b signaling of CD90^+^ stromal cells contribute to these ISCs promoting their effects. Vice versa, intestinal parasites, such as *G. lamblia* utilize their ADI system to deplete L-arg form the environment of the host and to hamper intestinal immune defense.

Abbreviations: ADI, arginine deiminase; Arg1, arginase isoform 1; IEC, intestinal epithelial cell; ISCs, intestinal stem cells; NOS2, inducible nitric oxide synthase; OTC, ornithine transcarbamylase; P-mTor, phosphorylated mammalian target of rapamycin; sIgA, secretory immunoglobulin A;

## Abbreviations

5-FU-: 5-fluoruracil
ACE2: angiotensin-converting enzyme 2
ADC: arginine decarboxylase
ADI: arginine deiminase
AdiC: arginine-agmatine antiporter
ADMA: asymmetric dimethylarginine
ADP: adenosine diphosphate
AO: arginine oxidase
ArcD: arginine-ornithine antiporter
Arg: arginase
Arg1: arginase isoform 1
argA: N-acetylglutamate synthase
argB: N-acetylglutamate kinase
argC: acetyl-y-glutamyl-phosphate reductase
argD: aceylornithine aminotransferase
argE: acetylornithine deacetylase
argF, argI: ornithine transcarbamyalase
argG: Argininosuccinate synthetase
argH: Argininosuccinate lyase
argJ: N-acetylglutamate synthetase
argR: arginine deiminase system associated receptor
argT: lysine-arginine-ornithine-binding periplasmic protein=
ASL: argininosuccinate lyase
Asl: argininosuccinate lyase
Ass: argininosuccinate synthase
AST: arginine succinyltransferase pathway
ATP: adenosine triphosphate
ATP7A: copper transporting ATPase
ATR: acid tolerance response
*C. albicans*: *Candida albicans*
*C. difficile*: *Clostridioides difficile*
*C. parvum*: *Cryptosporidium parvum*
*C. rodentium*: *Citrobacter rodentium*
CarA: Carbamoylphosphate synthase
CATs: cationic amino acid transpoters
CDI: *Clostridioides difficile* infection
CK: carbamate kinase
CO2: carbondioxide
Cps: carbamoylphosphate synthase
Cu2+: copper
DAO: diamine oxidase
DC: dendritic cell
di-GMP: dimeric guanosine monophosphate
DSS: dextran sodium sulfate
*E. coli*: *Escherichia coli*
EHEC: enterohemorrhagic *E. coli*
EPEC: enteropathogenic *E. coli*
*G. lamblia*: *Giardia lamblia*
GCN2: general control nonderepressible 2
GlcNAc: N-acetyl glucosamine
GPCRs: G-protein-coupled receptors
IBD: inflammatory bowel disease
IEC: intestinal epithelial cell
IL-: interleukin-
ILCs: innate lymphoid cells
ISCs: intestinal stem cells
L-arg: L-arginine
L-NMMA: n-monomethyl-1-arginine
LPS: lipopolysaccharide
MDSC: myeloid derived supressor cell
mTORC1: mechanistic target of rapamycin complex 1
NAO: N-acetyl-L-ornithine
NEC: necrotizing enterocolitis
NET: neutrophil extracellular trap
NH3: ammonia
NH4: ammonium
NK: natural killer cell
NO: nitric oxide
NOS: nitric oxide synthase
NOS2: inducible nitric oxide synthase
Oat: ornithine aminotransferase
OCT: ornithine carbamoyltransferase
ODC: ornithine decarboxylase
OTC: ornithine transcarbamoylase
OTU: operational taxonomic units
P-5-C: Pyrroline-5-carboxylate
P5cdh: Pyrroline-5-carboxylate dehydrogenase
P5cr: Pyrroline-5-carboxylate reductase
P5cs: Pyrroline-5-carboxylate synthase
Pi: phosphat inorganic
P-mTor: phosphorylated mechanistic target of rapamycin
Prodh: proline dehydrogenase
*S*.Tm: *Salmonella enterica* serovar Typhimurium
ScsC: periplasmic suppressor protein of copper sensitivity
SCV: *Salmonella* containing vacuole
SDMA: symmetric dimethylarginine
sIgA: secretory immunoglobulin A
T3SS: type 3 secretion system
SLC7: solute carrier 7 family
SPI: *Salmonella* pathogenicity island
T3SSs: syringe-like type III secretion systems
Tat: twin-arginine translocation
TcdA: toxin A
TcdB: toxin B
UC: ulcerative colitis
UDP-GlcNAc: uridine diphosphate N-acetylglucosamine
Wnt: wingless-type MMTV integration site family member

## Data Availability

All data analyzed during this study are included in this published article.

## References

[1] Schaefer K, Wagener J, Ames RM, Christou S, MacCallum DM, Bates S, Gow NAR. Three related enzymes in Candida albicans achieve arginine- and agmatine-dependent metabolism that is essential for growth and fungal virulence. mBio. 2020;11(4). doi:10.1128/mBio.01845-20. PMC7439472 32788384

[2] Slocum RD. Genes, enzymes and regulation of arginine biosynthesis in plants. Plant Physiol Biochem. 2005;43(8):729–31. doi:10.1016/j.plaphy.2005.06.007. 16122935

[3] Morris SM, Jr. Arginine metabolism revisited. J Nutr. 2016;146(12):2579S–2586S. doi:10.3945/jn.115.226621. 27934648

[4] Lu CD. Pathways and regulation of bacterial arginine metabolism and perspectives for obtaining arginine overproducing strains. Appl Microbiol Biotechnol. 2006;70(3):261–272. doi:10.1007/s00253-005-0308-z. 16432742

[5] Bogdan C. Nitric oxide synthase in innate and adaptive immunity: an update. Trends Immunol. 2015;36(3):161–178. doi:10.1016/j.it.2015.01.003. 25687683

[6] Morris SM, Jr. Enzymes of arginine metabolism. J Nutr. 2004;134(10):2743S–2747S; discussion 2765S-2767S. doi:10.1093/jn/134.10.2743S. 15465778

[7] Appleton J. Arginine: clinical potential of a semi-essential amino acid. Altern Med Rev. 2002;7:512–522. 12495375

[8] Marini JC, Agarwal U, Didelija IC. Dietary arginine requirements for growth are dependent on the rate of citrulline production in mice. J Nutr. 2015;145(6):1227–1231. doi:10.3945/jn.114.209668. 25855119 PMC4442117

[9] Wakabayashi Y, Jones ME. Pyrroline-5-carboxylate synthesis from glutamate by rat intestinal mucosa. J Biol Chem. 1983;258(6):3865–3872. doi:10.1016/S0021-9258(18)32747-9. 6131889

[10] Wakabayashi Y, Yamada E, Yoshida T, Takahashi H. Arginine becomes an essential amino acid after massive resection of rat small intestine. J Biol Chem. 1994;269(51):32667–32671. doi:10.1016/S0021-9258(18)31686-7. 7798273

[11] Windmueller HG, Spaeth AE. Source and fate of circulating citrulline. Am J Physiol. 1981;241(6):E473–480. doi:10.1152/ajpendo.1981.241.6.E473. 7325229

[12] Crenn P, Cynober L. Effect of intestinal resections on arginine metabolism: practical implications for nutrition support. Curr Opin Clin Nutr Metab Care. 2010;13(1):65–69. doi:10.1097/MCO.0b013e328333c1a8. 19915459

[13] Luiking YC, Poeze M, Ramsay G, Deutz NE. The role of arginine in infection and sepsis. JPEN J Parenter Enteral Nutr. 2005;29(1S):S70–74. doi:10.1177/01486071050290S1S70. 15709548

[14] Fritz JH, Adams JH. Arginine cools the inflamed gut. Infect Immun. 2013;81(10):3500–3502. doi:10.1128/IAI.00789-13. 23897606 PMC3811762

[15] Sender R, Fuchs S, Milo R. Are we really vastly outnumbered? Revisiting the ratio of bacterial to host cells in humans. Cell. 2016;164(3):337–340. doi:10.1016/j.cell.2016.01.013. 26824647

[16] Kayama H, Okumura R, Takeda K. Interaction between the microbiota, epithelia, and immune cells in the intestine. Annu Rev Immunol. 2020;38(1):23–48. doi:10.1146/annurev-immunol-070119-115104. 32340570

[17] Sommer F, Backhed F. Know your neighbor: microbiota and host epithelial cells interact locally to control intestinal function and physiology. Bioessays. 2016;38(5):455–464. doi:10.1002/bies.201500151. 26990415

[18] Wrage M, Kaltwasser J, Menge S, Mattner J. CD101 as an indicator molecule for pathological changes at the interface of host-microbiota interactions. Int J Med Microbiol. 2021;311(4):151497. doi:10.1016/j.ijmm.2021.151497. 33773220

[19] Miner-Williams WM, Moughan PJ. Intestinal barrier dysfunction: implications for chronic inflammatory conditions of the bowel. Nutr Res Rev. 2016;29(1):40–59. doi:10.1017/S0954422416000019. 27087106

[20] Pedersen HK, Gudmundsdottir V, Nielsen HB, Hyotylainen T, Nielsen T, Jensen BAH, Forslund K, Hildebrand F, Prifti E, Falony G, et al. Human gut microbes impact host serum metabolome and insulin sensitivity. Nature. 2016;535(7612):376–381. doi:10.1038/nature18646. 27409811

[21] Kinashi Y, Hase K. Partners in leaky gut syndrome: intestinal dysbiosis and autoimmunity. Front Immunol. 2021;12:673708. doi:10.3389/fimmu.2021.673708. 33968085 PMC8100306

[22] Lloyd-Price J, Arze C, Ananthakrishnan AN, Schirmer M, Avila-Pacheco J, Poon TW, Andrews E, Ajami NJ, Bonham KS, Brislawn CJ, et al. Multi-omics of the gut microbial ecosystem in inflammatory bowel diseases. Nature. 2019;569(7758):655–662. doi:10.1038/s41586-019-1237-9. 31142855 PMC6650278

[23] Franzosa EA, Sirota-Madi A, Avila-Pacheco J, Fornelos N, Haiser HJ, Reinker S, Vatanen T, Hall AB, Mallick H, McIver LJ, et al. Gut microbiome structure and metabolic activity in inflammatory bowel disease. Nature Microbiol. 2019;4(2):293–305. doi:10.1038/s41564-018-0306-4. 30531976 PMC6342642

[24] Singh K, Gobert AP, Coburn LA, Barry DP, Allaman M, Asim M, Luis PB, Schneider C, Milne GL, Boone HH, et al. Dietary arginine regulates severity of experimental colitis and affects the colonic microbiome. Front Cell Infect Microbiol. 2019;9:66. doi:10.3389/fcimb.2019.00066. 30972302 PMC6443829

[25] Bertrand J, Goichon A, Dechelotte P, Coeffier M. Regulation of intestinal protein metabolism by amino acids. Amino Acids. 2013;45(3):443–450. doi:10.1007/s00726-012-1325-8. 22643845

[26] Davis JS, Anstey NM. Is plasma arginine concentration decreased in patients with sepsis? A systematic review and meta-analysis. Crit Care Med. 2011;39(2):380–385. doi:10.1097/CCM.0b013e3181ffd9f7. 21150584

[27] Weiss SL, Haymond S, Ranaivo HR, Wang D, De Jesus VR, Chace DH, Wainwright MS. Evaluation of asymmetric dimethylarginine, arginine, and carnitine metabolism in pediatric sepsis. Pediatr Crit Care Med. 2012;13(4):e210–e218. doi:10.1097/PCC.0b013e318238b5cd. 22460770 PMC3392424

[28] Winkler MS, Nierhaus A, Rösler G, Lezius S, Harlandt O, Schwedhelm SE, Böger RH, Kluge S. Symmetrical (SDMA) and asymmetrical dimethylarginine (ADMA) in sepsis: high plasma levels as combined risk markers for sepsis survival. Crit Care. 2018;22(1):216. doi:10.1186/s13054-018-2090-1. 30231905 PMC6145330

[29] Luiking YC, Poeze M, Ramsay G, Deutz NE. Reduced citrulline production in sepsis is related to diminished de novo arginine and nitric oxide production. Am J Clin Nutr. 2009;89(1):142–152. doi:10.3945/ajcn.2007.25765. 19056593

[30] Kao CC, Bandi V, Guntupalli K, Wu M, Castillo L, Jahoor F. Arginine, citrulline and nitric oxide metabolism in sepsis. Clin Sci (Lond). 2009;117(1):23–30. doi:10.1042/CS20080444. 19105791

[31] Villalpando S, Gopal J, Balasubramanyam A, Bandi VP, Guntupalli K, Jahoor F. In vivo arginine production and intravascular nitric oxide synthesis in hypotensive sepsis. Am J Clin Nutr. 2006;84(1):197–203. doi:10.1093/ajcn/84.1.197. 16825696

[32] Davis JS, Yeo TW, Thomas JH, McMillan M, Darcy CJ, McNeil YR, Cheng AC, Celermajer DS, Stephens DP, Anstey NM, et al. Sepsis-associated microvascular dysfunction measured by peripheral arterial tonometry: an observational study. Crit Care. 2009;13(5):R155. doi:10.1186/cc8055. 19778457 PMC2784378

[33] Singh J, Lee Y, Kellum JA. A new perspective on NO pathway in sepsis and ADMA lowering as a potential therapeutic approach. Crit Care. 2022;26(1):246. doi:10.1186/s13054-022-04075-0. 35962414 PMC9373887

[34] Bronte V, Zanovello P. Regulation of immune responses by L-arginine metabolism. Nat Rev Immunol. 2005;5(8):641–654. doi:10.1038/nri1668. 16056256

[35] Boomer JS, To K, Chang KC, Takasu O, Osborne DF, Walton AH, Bricker TL, Jarman SD, Kreisel D, Krupnick AS, et al. Immunosuppression in patients who die of sepsis and multiple organ failure. JAMA. 2011;306(23):2594–2605. doi:10.1001/jama.2011.1829. 22187279 PMC3361243

[36] Daviaud F, Grimaldi D, Dechartres A, Charpentier J, Geri G, Marin N, Chiche JD, Cariou A, Mira JP, Pène F, et al. Timing and causes of death in septic shock. Ann Intensive Care. 2015;5(1):16. doi:10.1186/s13613-015-0058-8. 26092499 PMC4474967

[37] Venet F, Monneret G. Advances in the understanding and treatment of sepsis-induced immunosuppression. Nat Rev Nephrol. 2018;14(2):121–137. doi:10.1038/nrneph.2017.165. 29225343

[38] Costa KA, Soares ADN, Wanner SP, Santos RDGCD, Fernandes SOA, Martins FDS, Nicoli JR, Coimbra CC, Cardoso VN. L-arginine supplementation prevents increases in intestinal permeability and bacterial translocation in male swiss mice subjected to physical exercise under environmental heat stress. J Nutri. 2014;144(2):218–223. doi:10.3945/jn.113.183186. 24259555

[39] Viana ML, dos Santos RDGC, Generoso SDV, Nicoli JR, Martins FDS, Nogueira-Machado JA, Arantes RME, Correia MITD, Cardoso VN. The role of L-arginine-nitric oxide pathway in bacterial translocation. Amino Acids. 2013;45(5):1089–1096. doi:10.1007/s00726-013-1558-1. 23864434

[40] Becker RM, Wu G, Galanko JA, Chen W, Maynor AR, Bose CL, Rhoads JM. Reduced serum amino acid concentrations in infants with necrotizing enterocolitis. J Pediatr. 2000;137(6):785–793. doi:10.1067/mpd.2000.109145. 11113834

[41] Richir MC, Siroen MPC, van Elburg RM, Fetter WPF, Quik F, Nijveldt RJ, Heij HA, Smit BJ, Teerlink T, van Leeuwen PAM, et al. Low plasma concentrations of arginine and asymmetric dimethylarginine in premature infants with necrotizing enterocolitis. Br J Nutr. 2007;97(5):906–911. doi:10.1017/S0007114507669268. 17381965

[42] Zamora SA, Amin HJ, McMillan DD, Kubes P, Fick GH, Butzner JD, Parsons HG, Scott RB. Plasma L-arginine concentrations in premature infants with necrotizing enterocolitis. J Pediatr. 1997;131(2):226–232. doi:10.1016/s0022-3476(97)70158-6. 9290608

[43] Badurdeen S, Mulongo M, Berkley JA. Arginine depletion increases susceptibility to serious infections in preterm newborns. Pediatr Res. 2015;77(2):290–297. doi:10.1038/pr.2014.177. 25360828 PMC4335378

[44] Leung KT, Chan KYY, Ma TPY, Yu JWS, Tong JHM, Tam YH, Cheung HM, To KF, Lam HS, Lee KH, et al. Dysregulated expression of arginine metabolic enzymes in human intestinal tissues of necrotizing enterocolitis and response of CaCO2 cells to bacterial components. J Nutr Biochem. 2016;29:64–72. doi:10.1016/j.jnutbio.2015.10.010. 26895666

[45] Das P, Lahiri A, Lahiri A, Chakravortty D, Manchester M. Modulation of the arginase pathway in the context of microbial pathogenesis: a metabolic enzyme moonlighting as an immune modulator. PLoS Pathog. 2010;6(6):e1000899. doi:10.1371/journal.ppat.1000899. 20585552 PMC2887468

[46] Cuervo H, Guerrero NA, Carbajosa S, Beschin A, De Baetselier P, Gironès N, Fresno M. Myeloid-derived suppressor cells infiltrate the heart in acute trypanosoma cruzi infection. J Immunol. 2011;187(5):2656–2665. doi:10.4049/jimmunol.1002928. 21804013

[47] Stadelmann B, Hanevik K, Andersson MK, Bruserud O, Svard SG. The role of arginine and arginine-metabolizing enzymes during giardia – host cell interactions in vitro. BMC Microbiol. 2013;13(1):256. doi:10.1186/1471-2180-13-256. 24228819 PMC4225669

[48] Stadelmann B, Merino MC, Persson L, Svard SG, Zilberstein D. Arginine consumption by the intestinal parasite giardia intestinalis reduces proliferation of intestinal epithelial cells. PLoS One. 2012;7(9):e45325. doi:10.1371/journal.pone.0045325. 23028934 PMC3446895

[49] Ortega-Pierres MG, Arguello-Garcia R. Giardia duodenalis: role of secreted molecules as virulent factors in the cytotoxic effect on epithelial cells. Adv Parasitol. 2019;106:129–169. doi:10.1016/bs.apar.2019.07.003. 31630757

[50] Gobert AP, McGee DJ, Akhtar M, Mendz GL, Newton JC, Cheng Y, Mobley HLT, Wilson KT. Helicobacter pylori arginase inhibits nitric oxide production by eukaryotic cells: a strategy for bacterial survival. Proc Natl Acad Sci USA. 2001;98(24):13844–13849. doi:10.1073/pnas.241443798. 11717441 PMC61129

[51] Dai ZL, Li XL, Xi PB, Zhang J, Wu G, Zhu WY. Regulatory role for L-arginine in the utilization of amino acids by pig small-intestinal bacteria. Amino Acids. 2012;43(1):233–244. doi:10.1007/s00726-011-1067-z. 21928075

[52] Baier J, Gänsbauer M, Giessler C, Arnold H, Muske M, Schleicher U, Lukassen S, Ekici A, Rauh M, Daniel C, et al. Arginase impedes the resolution of colitis by altering the microbiome and metabolome. J Clin Invest. 2020;130(11):5703–5720. doi:10.1172/JCI126923. 32721946 PMC7598089

[53] Hong SK, Maltz BE, Coburn LA, Slaughter JC, Chaturvedi R, Schwartz DA, Wilson KT. Increased serum levels of L-arginine in ulcerative colitis and correlation with disease severity. Inflamm Bowel Dis. 2010;16(1):105–111. doi:10.1002/ibd.21035. 19637336 PMC2795785

[54] Coburn LA, Horst SN, Allaman MM, Brown CT, Williams CS, Hodges ME, Druce JP, Beaulieu DB, Schwartz DA, Wilson KT, et al. L-Arginine availability and metabolism is altered in ulcerative colitis. Inflamm Bowel Dis. 2016;22(8):1847–1858. doi:10.1097/MIB.0000000000000790. 27104830 PMC4956554

[55] Li JY, Guo YC, Zhou HF, Yue TT, Wang FX, Sun F, Wang WZ. Arginine metabolism regulates the pathogenesis of inflammatory bowel disease. Nutr Rev. 2022;81(5):578–586. doi:10.1093/nutrit/nuac070. PMC10086623 36040377

[56] Owczarek D, Cibor D, Mach T. Asymmetric dimethylarginine (ADMA), symmetric dimethylarginine (SDMA), arginine, and 8-iso-prostaglandin F2α (8-iso-PGF2α) level in patients with inflammatory bowel diseases. Inflamm Bowel Dis. 2010;16(1):52–57. doi:10.1002/ibd.20994. 19575355

[57] Alexander M, Ang QY, Nayak RR, Bustion AE, Sandy M, Zhang B, Upadhyay V, Pollard KS, Lynch SV, Turnbaugh PJ, et al. Human gut bacterial metabolism drives Th17 activation and colitis. Cell Host Microbe. 2022;30(1):17–30 e19. doi:10.1016/j.chom.2021.11.001. 34822777 PMC8785648

[58] Morgan XC, Tickle TL, Sokol H, Gevers D, Devaney KL, Ward DV, Reyes JA, Shah SA, LeLeiko N, Snapper SB, et al. Dysfunction of the intestinal microbiome in inflammatory bowel disease and treatment. Genome Biol. 2012;13(9):R79. doi:10.1186/gb-2012-13-9-r79. 23013615 PMC3506950

[59] Horowitz S, Binion DG, Nelson VM, Kanaa Y, Javadi P, Lazarova Z, Andrekopoulos C, Kalyanaraman B, Otterson MF, Rafiee P, et al. Increased arginase activity and endothelial dysfunction in human inflammatory bowel disease. Am J Physiol Gastrointest Liver Physiol. 2007;292(5):G1323–1336. doi:10.1152/ajpgi.00499.2006. 17218473

[60] Rees CA, Rostad CA, Mantus G, Anderson EJ, Chahroudi A, Jaggi P, Wrammert J, Ochoa JB, Ochoa A, Basu RK, et al. Altered amino acid profile in patients with SARS-CoV-2 infection. Proc Natl Acad Sci USA. 2021;118(25). doi:10.1073/pnas.2101708118. PMC8237604 34088793

[61] Reizine F, Lesouhaitier M, Gregoire M, Pinceaux K, Gacouin A, Maamar A, Painvin B, Camus C, Le Tulzo Y, Tattevin P, et al. SARS-CoV-2-induced ARDS associates with MDSC expansion, lymphocyte dysfunction, and arginine shortage. J Clin Immunol. 2021;41(3):515–525. doi:10.1007/s10875-020-00920-5. 33387156 PMC7775842

[62] Hasimi A, Dogan O, Serdar CC, Serdar MA. Association of serum ADMA, SDMA and L-NMMA concentrations with disease progression in COVID-19 patients. Biochem Med (Zagreb). 2023;33:010701. doi:10.11613/BM.2023.010701. 36627978 PMC9807234

[63] Haj AK, Hasan H, Raife TJ. Heritability of protein and metabolite biomarkers associated with COVID-19 severity: a metabolomics and proteomics analysis. Biomolecul. 2022;13(1):46. doi:10.3390/biom13010046. PMC9855380 36671431

[64] Drover JW, Dhaliwal R, Weitzel L, Wischmeyer PE, Ochoa JB, Heyland DK. Perioperative use of arginine-supplemented diets: a systematic review of the evidence. J Am Coll Surg. 2011;212(3):385–399e1. doi:10.1016/j.jamcollsurg.2010.10.016. 21247782

[65] Morris CR, Hamilton-Reeves J, Martindale RG, Sarav M, Ochoa Gautier JB. Acquired amino acid deficiencies: a focus on arginine and glutamine. Nutr Clin Pract. 2017;32(1_suppl):30S–47S. doi:10.1177/0884533617691250. 28388380

[66] Bronte V, Serafini P, Mazzoni A, Segal DM, Zanovello P. L-arginine metabolism in myeloid cells controls T-lymphocyte functions. Trends Immunol. 2003;24(6):302–306. doi:10.1016/s1471-4906(03)00132-7. 12810105

[67] Tattoli I, Sorbara MT, Vuckovic D, Ling A, Soares F, Carneiro LM, Yang C, Emili A, Philpott D, Girardin S, et al. Amino acid starvation induced by invasive bacterial pathogens triggers an innate host defense program. Cell Host Microbe. 2012;11(6):563–575. doi:10.1016/j.chom.2012.04.012. 22704617

[68] Krzystek-Korpacka M, Fleszar MG, Bednarz-Misa I, Lewandowski Ł, Szczuka I, Kempiński R, Neubauer K. Transcriptional and metabolomic analysis of L-Arginine/nitric oxide pathway in inflammatory bowel disease and its association with local inflammatory and angiogenic response: preliminary findings. IJMS. 2020;21:1641. doi:10.3390/ijms21051641. 32121248 PMC7084352

[69] Cyr AR, Huckaby LV, Shiva SS, Zuckerbraun BS. Nitric oxide and endothelial dysfunction. Crit Care Clin. 2020;36(2):307–321. doi:10.1016/j.ccc.2019.12.009. 32172815 PMC9015729

[70] Guslandi M. Nitric oxide and inflammatory bowel diseases. Eur J Clin Invest. 1998;28(11):904–907. doi:10.1046/j.1365-2362.1998.00377.x. 9824433

[71] Wang X, Sadeghirad B, Morgan RL, Zeratkaar D, Chang Y, Crandon HN, Couban R, Foroutan F, Florez ID. Amino acids for the prevention of mortality and morbidity in preterm infants: a systematic review and network meta-analysis. Sci Rep. 2022;12(1):18333. doi:10.1038/s41598-022-21318-w. 36316436 PMC9622873

[72] Szwed A, Kim E, Jacinto E. Regulation and metabolic functions of mTORC1 and mTORC2. Physiol Rev. 2021;101(3):1371–1426. doi:10.1152/physrev.00026.2020.s 33599151 PMC8424549

[73] Zhuang Y, Wang XX, He J, He S, Yin Y. Recent advances in understanding of amino acid signaling to mTORC1 activation. Front Biosci. 2019;24(5):971–982. doi:10.2741/4762. 30844724

[74] Halaby MJ, Hezaveh K, Lamorte S, Ciudad MT, Kloetgen A, MacLeod BL, Guo M, Chakravarthy A, Medina TDS, Ugel S, et al. GCN2 drives macrophage and MDSC function and immunosuppression in the tumor microenvironment. Sci Immunol. 2019;4(42). doi:10.1126/sciimmunol.aax8189. PMC7201901 31836669

[75] Averous J, Lambert-Langlais S, Mesclon F, Carraro V, Parry L, Jousse C, Bruhat A, Maurin AC, Pierre P, Proud CG, et al. GCN2 contributes to mTORC1 inhibition by leucine deprivation through an ATF4 independent mechanism. Sci Rep. 2016;6(1):27698. doi:10.1038/srep27698. 27297692 PMC4906353

[76] Jungnickel KEJ, Parker JL, Newstead S. Structural basis for amino acid transport by the CAT family of SLC7 transporters. Nat Commun. 2018;9(1):550. doi:10.1038/s41467-018-03066-6. 29416041 PMC5803215

[77] Wu G, Bazer FW, Satterfield MC, Gilbreath KR, Posey EA, Sun Y. L-Arginine nutrition and metabolism in ruminants. Adv Exp Med Biol. 2022;1354:177–206. doi:10.1007/978-3-030-85686-1_10. 34807443

[78] de Jonge WJ, Hallemeesch MM, Kwikkers KL, Ruijter JM, de Gier-de Vries C, van Roon MA, Meijer AJ, Marescau B, De Deyn PP, Deutz NE, et al. Overexpression of arginase I in enterocytes of transgenic mice elicits a selective arginine deficiency and affects skin, muscle, and lymphoid development. Am J Clin Nutr. 2002;76(1):128–140. doi:10.1093/ajcn/76.1.128. 12081826

[79] Zheng L, Kelly CJ, Colgan SP. Physiologic hypoxia and oxygen homeostasis in the healthy intestine. A review in the theme: cellular responses to hypoxia. Am J Physiol Cell Physiol. 2015;309(6):C350–360. doi:10.1152/ajpcell.00191.2015. 26179603 PMC4572369

[80] Boger RH. The pharmacodynamics of L-arginine. J Nutr. 2007;137(6):1650S–1655S. doi:10.1093/jn/137.6.1650S. 17513442

[81] Hernandez VM, Arteaga A, Dunn MF. Diversity, properties and functions of bacterial arginases. FEMS Microbiol Rev. 2021;45(6). doi:10.1093/femsre/fuab034. 34160574

[82] Menezes-Garcia Z, Kumar A, Zhu W, Winter SE, Sperandio V. L-Arginine sensing regulates virulence gene expression and disease progression in enteric pathogens. Proc Natl Acad Sci USA. 2020;117(22):12387–12393. doi:10.1073/pnas.1919683117. 32409599 PMC7275701

[83] Casiano-Colon A, Marquis RE. Role of the arginine deiminase system in protecting oral bacteria and an enzymatic basis for acid tolerance. Appl Environ Microbiol. 1988;54(6):1318–1324. doi:10.1128/aem.54.6.1318-1324.1988. 2843090 PMC202656

[84] Schofield PJ, Costello M, Edwards MR, O’Sullivan WJ. The arginine dihydrolase pathway is present in giardia intestinalis. Int J Parasitol. 1990;20(5):697–699. doi:10.1016/0020-7519(90)90133-8. 2228433

[85] Abolins S, King EC, Lazarou L, Weldon L, Hughes L, Drescher P, Raynes JG, Hafalla JCR, Viney ME, Riley EM, et al. The comparative immunology of wild and laboratory mice, Mus musculus domesticus. Nat Commun. 2017;8(1):14811. doi:10.1038/ncomms14811. 28466840 PMC5418598

[86] Ilgu H, Jeckelmann JM, Gapsys V, Ucurum Z, de Groot BL, Fotiadis D. Insights into the molecular basis for substrate binding and specificity of the wild-type L-arginine/agmatine antiporter AdiC. Proc Natl Acad Sci USA. 2016;113(37):10358–10363. doi:10.1073/pnas.1605442113. 27582465 PMC5027449

[87] Ghazisaeedi F, Meens J, Hansche B, Maurischat S, Schwerk P, Goethe R, Wieler LH, Fulde M, Tedin K. A virulence factor as a therapeutic: the probiotic enterococcus faecium SF68 arginine deiminase inhibits innate immune signaling pathways. Gut Microbes. 2022;14(1):2106105. doi:10.1080/19490976.2022.2106105. 35921516 PMC9351580

[88] Kawatra A, Dhankhar R, Gulati P. Microbial arginine deiminase: a multifaceted green catalyst in biomedical sciences. Int J Biol Macromol. 2022;196:151–162. doi:10.1016/j.ijbiomac.2021.12.015. 34920062

[89] Dave K, Ahuja M, Jayashri TN, Sirola RB, Punekar NS. A novel selectable marker based on aspergillus niger arginase expression. Enzyme Microb Technol. 2012;51(1):53–58. doi:10.1016/j.enzmictec.2012.04.001. 22579391

[90] Davis RH. Compartmental and regulatory mechanisms in the arginine pathways of neurospora crassa and Saccharomyces cerevisiae. Microbiol Rev. 1986;50(3):280–313. doi:10.1128/mr.50.3.280-313.1986. 2945985 PMC373072

[91] Nakamura A, Ooga T, Matsumoto M. Intestinal luminal putrescine is produced by collective biosynthetic pathways of the commensal microbiome. Gut Microbes. 2019;10(2):159–171. doi:10.1080/19490976.2018.1494466. 30183487 PMC6546329

[92] Kurihara S. Polyamine metabolism and transport in gut microbes. Biosci Biotechnol Biochem. 2022;86:957–966. doi:10.1093/bbb/zbac080. 35648468

[93] Grimes JM, Khan S, Badeaux M, Rao RM, Rowlinson SW, Carvajal RD. Arginine depletion as a therapeutic approach for patients with COVID-19. Int J Infect Dis. 2021;102:566–570. doi:10.1016/j.ijid.2020.10.100. 33160064 PMC7641537

[94] Gogoi M, Datey A, Wilson KT, Chakravortty D. Dual role of arginine metabolism in establishing pathogenesis. Curr Opin Microbiol. 2016;29:43–48. doi:10.1016/j.mib.2015.10.005. 26610300 PMC4755812

[95] Galan JE, Lara-Tejero M, Marlovits TC, Wagner S. Bacterial type III secretion systems: specialized nanomachines for protein delivery into target cells. Annu Rev Microbiol. 2014;68(1):415–438. doi:10.1146/annurev-micro-092412-155725. 25002086 PMC4388319

[96] Wagner S, Grin I, Malmsheimer S, Singh N, Torres-Vargas CE, Westerhausen S. Bacterial type III secretion systems: a complex device for the delivery of bacterial effector proteins into eukaryotic host cells. FEMS Microbiol Lett. 2018;365(19). doi:10.1093/femsle/fny201. PMC6140923 30107569

[97] Araujo-Garrido JL, Bernal-Bayard J, Ramos-Morales F. Type III secretion effectors with arginine N-Glycosyltransferase activity. Microorgan. 2020;8(3):357. doi:10.3390/microorganisms8030357. PMC7142665 32131463

[98] Schaffler H, Breitruck A. Clostridium difficile - from colonization to infection. Front Microbiol. 2018;9:646. doi:10.3389/fmicb.2018.00646. 29692762 PMC5902504

[99] Anderson A, Click B, Ramos-Rivers C, Cheng D, Babichenko D, Koutroubakis IE, Hashash JG, Schwartz M, Swoger J, Barrie AM, et al. Lasting impact of clostridium difficile infection in inflammatory bowel disease: a propensity score matched analysis. Inflamm Bowel Dis. 2017;23(12):2180–2188. doi:10.1097/MIB.0000000000001251. 29084081 PMC5685936

[100] Dai C, Jiang M, Sun MJ. The impact of clostridium difficile infection on mortality in patients with inflammatory bowel disease. J Clin Gastroenterol. 2019;53(2):155. doi:10.1097/MCG.0000000000000996. 29369236

[101] Bossuyt P, Verhaegen J, Van Assche G, Rutgeerts P, Vermeire S. Increasing incidence of clostridium difficile-associated diarrhea in inflammatory bowel disease. J Crohns Colitis. 2009;3(1):4–7. doi:10.1016/j.crohns.2008.09.003. 21172241

[102] Clayton EM, Rea MC, Shanahan F, Quigley EMM, Kiely B, Hill C, Ross RP. The vexed relationship between clostridium difficile and inflammatory bowel disease: an assessment of carriage in an outpatient setting among patients in remission. Am J Gastroenterol. 2009;104(5):1162–1169. doi:10.1038/ajg.2009.4. 19319128

[103] Battaglioli EJ, Hale VL, Chen J, Jeraldo P, Ruiz-Mojica C, Schmidt BA, Rekdal VM, Till LM, Huq L, Smits SA, et al. Clostridioides difficile uses amino acids associated with gut microbial dysbiosis in a subset of patients with diarrhea. Sci Transl Med. 2018;10(464). doi:10.1126/scitranslmed.aam7019. PMC6537101 30355801

[104] Pruss KM, Enam F, Battaglioli E, DeFeo M, Diaz OR, Higginbottom SK, Fischer CR, Hryckowian AJ, Van Treuren W, Dodd D, et al. Oxidative ornithine metabolism supports non-inflammatory C. difficile colonization. Nat Metab. 2022;4(1):19–28. doi:10.1038/s42255-021-00506-4. 34992297 PMC8803604

[105] Karasawa T, Maegawa T, Nojiri T, Yamakawa K, Nakamura S. Effect of arginine on toxin production by clostridium difficile in defined medium. Microbiol Immunol. 1997;41(8):581–585. doi:10.1111/j.1348-0421.1997.tb01895.x. 9310936

[106] Smith AB, Jenior ML, Keenan O, Hart JL, Specker J, Abbas A, Rangel PC, Di C, Green J, Bustin KA, et al. Enterococci enhance clostridioides difficile pathogenesis. Nature. 2022;611(7937):780–786. doi:10.1038/s41586-022-05438-x. 36385534 PMC9691601

[107] Sokol H, Jegou S, McQuitty C, Straub M, Leducq V, Landman C, Kirchgesner J, Le Gall G, Bourrier A, Nion-Larmurier I, et al. Specificities of the intestinal microbiota in patients with inflammatory bowel disease and clostridium difficile infection. Gut Microbes. 2018;9(1):55–60. doi:10.1080/19490976.2017.1361092. 28786749 PMC5914915

[108] Collins JW, Keeney KM, Crepin VF, Rathinam VAK, Fitzgerald KA, Finlay BB, Frankel G. Citrobacter rodentium: infection, inflammation and the microbiota. Nat Rev Microbiol. 2014;12(9):612–623. doi:10.1038/nrmicro3315. 25088150

[109] Pifer R, Russell RM, Kumar A, Curtis MM, Sperandio V. Redox, amino acid, and fatty acid metabolism intersect with bacterial virulence in the gut. Proc Natl Acad Sci USA. 2018;115(45):E10712–E10719. doi:10.1073/pnas.1813451115. 30348782 PMC6233112

[110] Canonaco F, Schlattner U, Wallimann T, Sauer U. Functional expression of arginine kinase improves recovery from pH stress of escherichia coli. Biotechnol Lett. 2003;25(13):1013–1017. doi:10.1023/a:1024172518062. 12889807

[111] Nicholson B, Manner CK, Kleeman J, MacLeod CL. Sustained nitric oxide production in macrophages requires the arginine transporter CAT2. J Biol Chem. 2001;276(19):15881–15885. doi:10.1074/jbc.M010030200. 11278602

[112] Chaturvedi R, Asim M, Hoge S, Lewis ND, Singh K, Barry DP, de Sablet T, Piazuelo MB, Sarvaria AR, Cheng Y, et al. Polyamines impair immunity to helicobacter pylori by inhibiting L-Arginine uptake required for nitric oxide production. Gastroenterol. 2010;139(5):1686–1698. doi:10.1053/j.gastro.2010.06.060. PMC2967614 20600019

[113] Singh K, Coburn LA, Barry DP, Asim M, Scull BP, Allaman MM, Lewis ND, Washington MK, Rosen MJ, Williams CS, et al. Deletion of cationic amino acid transporter 2 exacerbates dextran sulfate sodium colitis and leads to an IL-17-predominant T cell response. Am J Physiol Gastrointest Liver Physiol. 2013;305(3):G225–240. doi:10.1152/ajpgi.00091.2013. 23703655 PMC3742860

[114] Singh K, Al-Greene NT, Verriere TG, Coburn LA, Asim M, Barry DP, Allaman MM, Hardbower DM, Delgado AG, Piazuelo MB, et al. The L-Arginine transporter solute carrier family 7 member 2 mediates the immunopathogenesis of attaching and effacing bacteria. PLoS Pathog. 2016;12(10):e1005984. doi:10.1371/journal.ppat.1005984. 27783672 PMC5081186

[115] Otake T, Fujimoto M, Hoshino Y, Ishihara T, Haneda T, Okada N, Miki T. Twin-arginine translocation system is involved in citrobacter rodentium fitness in the intestinal tract. Infect Immun. 2020;88(3). doi:10.1128/IAI.00892-19. PMC7035917 31818958

[116] Lee PA, Tullman-Ercek D, Georgiou G. The bacterial twin-arginine translocation pathway. Annu Rev Microbiol. 2006;60(1):373–395. doi:10.1146/annurev.micro.60.080805.142212. 16756481 PMC2654714

[117] Fujimoto M, Goto R, Hirota R, Ito M, Haneda T, Okada N, Miki T. Tat-exported peptidoglycan amidase-dependent cell division contributes to salmonella typhimurium fitness in the inflamed gut. PLoS Pathog. 2018;14(10):e1007391. doi:10.1371/journal.ppat.1007391. 30379938 PMC6231687

[118] Kaiser P, Diard M, Stecher B, Hardt WD. The streptomycin mouse model for salmonella diarrhea: functional analysis of the microbiota, the pathogen’s virulence factors, and the host’s mucosal immune response. Immunol Rev. 2012;245(1):56–83. doi:10.1111/j.1600-065X.2011.01070.x. 22168414

[119] Hapfelmeier S, Hardt WD. A mouse model for S. typhimurium-induced enterocolitis. Trends Microbiol. 2005;13(10):497–503. doi:10.1016/j.tim.2005.08.008. 16140013

[120] Giordano NP, Mettlach JA, Dalebroux ZD, Raffatellu M. Conserved tandem arginines for PbgA/YejM allow salmonella typhimurium to regulate LpxC and control lipopolysaccharide biogenesis during infection. Infect Immun. 2022;90(2):e0049021. doi:10.1128/IAI.00490-21. 34780276 PMC8853683

[121] El Qaidi S, Scott NE, Hays MP, Hardwidge PR. Arginine glycosylation regulates UDP-GlcNAc biosynthesis in salmonella enterica. Sci Rep. 2022;12(1):5293. doi:10.1038/s41598-022-09276-9. 35351940 PMC8964723

[122] Xue J, Huang Y, Zhang H, Hu J, Pan X, Peng T, Lv J, Meng K, Li S. Arginine GlcNAcylation and activity regulation of PhoP by a type III secretion system effector in salmonella. Front Microbiol. 2021;12:825743. doi:10.3389/fmicb.2021.825743. 35126337 PMC8811161

[123] Park JB, Kim YH, Yoo Y, Kim J, Jun SH, Cho JW, El Qaidi S, Walpole S, Monaco S, García-García AA, et al. Structural basis for arginine glycosylation of host substrates by bacterial effector proteins. Nat Commun. 2018;9(1):4283. doi:10.1038/s41467-018-06680-6. 30327479 PMC6191443

[124] Newson JM, Scott N, Yeuk Wah Chung I, Wong Fok Lung T, Giogha C, Gan J, Wang N, Strugnell RA, Brown NF, Cygler M, et al. Salmonella effectors SseK1 and SseK3 target death domain proteins in the TNF and TRAIL signaling pathways. Mol Cell Proteom. 2019;18(6):1138–1156. doi:10.1074/mcp.RA118.001093. PMC6553940 30902834

[125] Scott NE, Giogha C, Pollock GL, Kennedy CL, Webb AI, Williamson NA, Pearson JS, Hartland EL. The bacterial arginine glycosyltransferase effector NleB preferentially modifies fas-associated death domain protein (FADD). J Biol Chem. 2017;292(42):17337–17350. doi:10.1074/jbc.M117.805036. 28860194 PMC5655511

[126] Schmid J, Heider D, Wendel NJ, Sperl N, Sieber V. Bacterial glycosyltransferases: challenges and opportunities of a highly diverse enzyme class toward tailoring natural products. Front Microbiol. 2016;7:182. doi:10.3389/fmicb.2016.00182. 26925049 PMC4757703

[127] Mills E, Petersen E, Kulasekara BR, Miller SI. A direct screen for c-di-GMP modulators reveals a salmonella typhimurium periplasmic ʟ-arginine–sensing pathway. Sci Signal. 2015;8(380):ra57. doi:10.1126/scisignal.aaa1796. 26060330

[128] Yucel B, Robinson GK, Shepherd M. The copper-responsive ScsC protein of salmonella promotes intramacrophage survival and interacts with the arginine sensor artI. FEBS J. 2020;287(17):3827–3840. doi:10.1111/febs.15285. 32153092

[129] Guerra PR, Liu G, Lemire S, Nawrocki A, Kudirkiene E, Møller-Jensen J, Olsen JE, Jelsbak L. Polyamine depletion has global effects on stress and virulence gene expression and affects HilA translation in salmonella enterica serovar typhimurium. Res Microbiol. 2020;171(3–4):143–152. doi:10.1016/j.resmic.2019.12.001. 31991172

[130] Deka G, Bisht S, Savithri HS, Murthy MRN. Comparative structural and enzymatic studies on salmonella typhimurium diaminopropionate ammonia lyase reveal its unique features. J Struct Biol. 2018;202(2):118–128. doi:10.1016/j.jsb.2017.12.012. 29294403

[131] Kieboom J, Abee T. Arginine-dependent acid resistance in salmonella enterica serovar typhimurium. J Bacteriol. 2006;188(15):5650–5653. doi:10.1128/JB.00323-06. 16855258 PMC1540025

[132] Choi Y, Choi J, Groisman EA, Kang DH, Shin D, Ryu S. Expression of STM4467-encoded arginine deiminase controlled by the STM4463 regulator contributes to salmonella enterica serovar typhimurium virulence. Infect Immun. 2012;80(12):4291–4297. doi:10.1128/IAI.00880-12. 23006851 PMC3497419

[133] Einarsson E, Ma’ayeh S, Svard SG. An up-date on giardia and giardiasis. Curr Opin Microbiol. 2016;34:47–52. doi:10.1016/j.mib.2016.07.019. 27501461

[134] Fink MY, Singer SM. The intersection of immune responses, microbiota, and pathogenesis in giardiasis. Trends Parasitol. 2017;33(11):901–913. doi:10.1016/j.pt.2017.08.001. 28830665 PMC5660942

[135] Barash NR, Maloney JG, Singer SM, Dawson SC, Appleton JA. Giardia alters commensal microbial diversity throughout the murine gut. Infect Immun. 2017;85(6). doi:10.1128/IAI.00948-16. PMC5442636 28396324

[136] Barash NR, Nosala C, Pham JK, McInally SG, Gourguechon S, McCarthy-Sinclair B, Dawson SC. Giardia colonizes and encysts in high-density foci in the murine small intestine. mSphere. 2017;2(3). doi:10.1128/mSphere.00343-16. PMC5480036 28656177

[137] Halliez MC, Buret AG. Extra-intestinal and long term consequences of giardia duodenalis infections. World J Gastroenterol. 2013;19(47):8974–8985. doi:10.3748/wjg.v19.i47.8974. 24379622 PMC3870550

[138] Oberhuber G, Kastner N, Stolte M. Giardiasis: a histologic analysis of 567 cases. Scand J Gastroenterol. 1997;32(1):48–51. doi:10.3109/00365529709025062. 9018766

[139] Dann SM, Le CHY, Hanson EM, Ross MC, Eckmann L. Giardia infection of the small intestine induces chronic colitis in genetically susceptible hosts. J Immunol. 2018;201(2):548–559. doi:10.4049/jimmunol.1700824. 29898958 PMC7351291

[140] Ringqvist E, Palm JED, Skarin H, Hehl AB, Weiland M, Davids BJ, Reiner DS, Griffiths WJ, Eckmann L, Gillin FD, et al. Release of metabolic enzymes by giardia in response to interaction with intestinal epithelial cells. Mol Biochem Parasitol. 2008;159(2):85–91. doi:10.1016/j.molbiopara.2008.02.005. 18359106 PMC3658456

[141] Maloney J, Keselman A, Li E, Singer SM. Macrophages expressing arginase 1 and nitric oxide synthase 2 accumulate in the small intestine during giardia lamblia infection. Microbes Infect. 2015;17(6):462–467. doi:10.1016/j.micinf.2015.03.006. 25797399 PMC4461514

[142] Eckmann L, Laurent F, Langford TD, Hetsko ML, Smith JR, Kagnoff MF, Gillin FD. Nitric oxide production by human intestinal epithelial cells and competition for arginine as potential determinants of host defense against the lumen-dwelling pathogen giardia lamblia. J Immunol. 2000;164(3):1478–1487. doi:10.4049/jimmunol.164.3.1478. 10640765

[143] Checkley W, White AC, Jaganath D, Arrowood MJ, Chalmers RM, Chen XM, Fayer R, Griffiths JK, Guerrant RL, Hedstrom L, et al. A review of the global burden, novel diagnostics, therapeutics, and vaccine targets for cryptosporidium. Lancet Infect Dis. 2015;15(1):85–94. doi:10.1016/S1473-3099(14)70772-8. 25278220 PMC4401121

[144] de Graaf DC, Vanopdenbosch E, Ortega-Mora LM, Abbassi H, Peeters JE. A review of the importance of cryptosporidiosis in farm animals. Int J Parasitol. 1999;29(8):1269–1287. doi:10.1016/s0020-7519(99)00076-4. 10576578 PMC7127282

[145] Rahman SU, Gong H, Mi R, Huang Y, Han X, Chen Z. Chitosan protects immunosuppressed mice against cryptosporidium parvum infection through TLR4/STAT1 signaling pathways and gut microbiota modulation. Front Immunol. 2021;12:784683. doi:10.3389/fimmu.2021.784683. 35095858 PMC8795679

[146] Rahman SU, Zhou K, Zhou S, Sun T, Mi R, Huang Y, Han X, Gong H, Chen Z. Curcumin mitigates cryptosporidium parvum infection through modulation of gut microbiota and innate immune-related genes in immunosuppressed neonatal mice. Microb Pathog. 2022;164:105424. doi:10.1016/j.micpath.2022.105424. 35092833

[147] Carey MA, Medlock GL, Alam M, Kabir M, Uddin MJ, Nayak U, Papin J, Faruque ASG, Haque R, Petri WA, et al. Megasphaera in the stool microbiota is negatively associated with diarrheal cryptosporidiosis. Clin Infect Dis. 2021;73(6):e1242–e1251. doi:10.1093/cid/ciab207. 33684930 PMC8442784

[148] VanDussen KL, Funkhouser-Jones LJ, Akey ME, Schaefer DA, Ackman K, Riggs MW, Stappenbeck TS, Sibley LD. Neonatal mouse gut metabolites influence cryptosporidium parvum infection in intestinal epithelial cells. mBio. 2020;11(6). doi:10.1128/mBio.02582-20. PMC7773987 33323514

[149] Russler-Germain EV, Jung J, Miller AT, Young S, Yi J, Wehmeier A, Fox LE, Monte KJ, Chai JN, Kulkarni DH, et al. Commensal cryptosporidium colonization elicits a cDC1-dependent Th1 response that promotes intestinal homeostasis and limits other infections. Immunity. 2021;54(11):2547–2564 e2547. doi:10.1016/j.immuni.2021.10.002. 34715017 PMC8716016

[150] Leitch GJ, He Q, Kozel TR. Reactive nitrogen and oxygen species ameliorate experimental cryptosporidiosis in the neonatal BALB/c mouse model. Infect Immun. 1999;67(11):5885–5891. doi:10.1128/IAI.67.11.5885-5891.1999. 10531244 PMC96970

[151] Gookin JL, Duckett LL, Armstrong MU, Stauffer SH, Finnegan CP, Murtaugh MP, Argenzio RA. Nitric oxide synthase stimulates prostaglandin synthesis and barrier function in C. parvum-infected porcine ileum. Am J Physiol Gastrointest Liver Physiol. 2004;287(3):G571–581. doi:10.1152/ajpgi.00413.2003. 15155179

[152] Argenzio RA, Lecce J, Powell DW. Prostanoids inhibit intestinal NaCl absorption in experimental porcine cryptosporidiosis. Gastroenterol. 1993;104(2):440–447. doi:10.1016/0016-5085(93)90412-6. 8425686

[153] Cole J, Blikslager A, Hunt E, Gookin J, Argenzio R. Cyclooxygenase blockade and exogenous glutamine enhance sodium absorption in infected bovine ileum. Am J Physiol Gastrointest Liver Physiol. 2003;284(3):G516–524. doi:10.1152/ajpgi.00172.2002. 12466144

[154] Gookin JL, Stauffer SH, Stone MR. Induction of arginase II by intestinal epithelium promotes the uptake of L-Arginine from the lumen of cryptosporidium parvum–infected porcine ileum. J Pediatr Gastroenterol Nutr. 2008;47(4):417–427. doi:10.1097/MPG.0b013e31816f6c02. 18852633 PMC3685577

[155] Azevedo OG, Bolick DT, Roche JK, Pinkerton RF, Lima AAM, Vitek MP, Warren CA, Oriá RB, Guerrant RL. Apolipoprotein E plays a key role against cryptosporidial infection in transgenic undernourished mice. PLoS One. 2014;9(2):e89562. doi:10.1371/journal.pone.0089562. 24586873 PMC3938486

[156] Mennigen R, Bieganski T, Elbers A, Kusche J. The histamine-diamine oxidase system and mucosal proliferation under the influence of aminoguanidine and seventy percent resection of the rat small intestine. Agents Actions. 1989;27(1–2):221–223. doi:10.1007/BF02222245. 2501973

[157] Miyoshi J, Miyamoto H, Goji T, Taniguchi T, Tomonari T, Sogabe M, Kimura T, Kitamura S, Okamoto K, Fujino Y, et al. Serum diamine oxidase activity as a predictor of gastrointestinal toxicity and malnutrition due to anticancer drugs. J Gastroenterol Hepatol. 2015;30(11):1582–1590. doi:10.1111/jgh.13004. 25968084

[158] Namikawa T, Fukudome I, Kitagawa H, Okabayashi T, Kobayashi M, Hanazaki K. Plasma diamine oxidase activity is a useful biomarker for evaluating gastrointestinal tract toxicities during chemotherapy with oral fluorouracil anti-cancer drugs in patients with gastric cancer. Oncology. 2012;82(3):147–152. doi:10.1159/000336799. 22433290

[159] Fukuda T, Tsukano K, Nakatsuji H, Suzuki K. Plasma diamine oxidase activity decline with diarrhea severity in calves indicating systemic dysfunction related to intestinal mucosal damage. Res Vet Sci. 2019;126:127–130. doi:10.1016/j.rvsc.2019.08.027. 31479828

[160] Tsukano K, Lakritz J, Suzuki K. Plasma amino acid status is useful for understanding intestinal mucosal damage in calves with cryptosporidiosis. Amino Acids. 2020;52(10):1459–1464. doi:10.1007/s00726-020-02904-6. 33090265

[161] Meingast CL, Joshi PU, Turpeinen DG, Xu X, Holstein M, Feroz H, Ranjan S, Ghose S, Li ZJ, Heldt CL, et al. Physiochemical properties of enveloped viruses and arginine dictate inactivation. Biotechnol J. 2021;16(7):e2000342. doi:10.1002/biot.202000342. 33877739

[162] Ikeda K, Yamasaki H, Minami S, Suzuki Y, Tsujimoto K, Sekino Y, IRIE H, Arakawa T, Koyama AH. Arginine inactivates human herpesvirus 2 and inhibits genital herpesvirus infection. Int J Mol Med. 2012;30(6):1307–1312. doi:10.3892/ijmm.2012.1149. 23042569

[163] Park JH, Kang I, Kim HC, Lee Y, Lee SK, Lee HK. Obesity enhances antiviral immunity in the genital mucosa through a microbiota-mediated effect on γδ T cells. Cell Rep. 2022;41(6):111594. doi:10.1016/j.celrep.2022.111594. 36351403

[164] Adiliaghdam F, Amatullah H, Digumarthi S, Saunders TL, Rahman RU, Wong LP, Sadreyev R, Droit L, Paquette J, Goyette P, et al. Human enteric viruses autonomously shape inflammatory bowel disease phenotype through divergent innate immunomodulation. Sci Immunol. 2022;7(70):eabn6660. doi:10.1126/sciimmunol.abn6660. 35394816 PMC9416881

[165] Norman JM, Handley S, Baldridge M, Droit L, Liu C, Keller B, Kambal A, Monaco C, Zhao G, Fleshner P, et al. Disease-specific alterations in the enteric virome in inflammatory bowel disease. Cell. 2015;160(3):447–460. doi:10.1016/j.cell.2015.01.002. 25619688 PMC4312520

[166] Ungaro F, Massimino L, Furfaro F, Rimoldi V, Peyrin-Biroulet L, D’Alessio S, Danese S. Metagenomic analysis of intestinal mucosa revealed a specific eukaryotic gut virome signature in early-diagnosed inflammatory bowel disease. Gut Microbes. 2019;10(2):149–158. doi:10.1080/19490976.2018.1511664. 30252582 PMC6546319

[167] Booth CM, Matukas LM, Tomlinson GA, Rachlis AR, Rose DB, Dwosh HA, Walmsley SL, Mazzulli T, Avendano M, Derkach P, et al. Clinical features and short-term outcomes of 144 patients with SARS in the greater Toronto area. JAMA. 2003;289(21):2801–2809. doi:10.1001/jama.289.21.JOC30885. 12734147

[168] Cheung KS, Hung IFN, Chan PPY, Lung KC, Tso E, Liu R, Ng YY, Chu MY, Chung TWH, Tam AR, et al. Gastrointestinal manifestations of SARS-CoV-2 infection and virus load in fecal samples from a Hong Kong cohort: systematic review and meta-analysis. Gastroenterol. 2020;159(1):81–95. doi:10.1053/j.gastro.2020.03.065. PMC7194936 32251668

[169] Zhou P, Yang XL, Wang XG, Hu B, Zhang L, Zhang W, Si HR, Zhu Y, Li B, Huang CL, et al. A pneumonia outbreak associated with a new coronavirus of probable bat origin. Nature. 2020;579(7798):270–273. doi:10.1038/s41586-020-2012-7. 32015507 PMC7095418

[170] Wrapp D, Wang N, Corbett KS, Goldsmith JA, Hsieh CL, Abiona O, Graham BS, McLellan JS. Cryo-EM structure of the 2019-nCov spike in the prefusion conformation. Sci. 2020;367(6483):1260–1263. doi:10.1126/science.abb2507. PMC7164637 32075877

[171] Patankar JV, Chiriac MT, Lehmann M, Kühl AA, Atreya R, Becker C, Gonzalez-Acera M, Schmitt H, Gamez-Belmonte R, Mahapatro M, et al. Severe acute respiratory syndrome coronavirus 2 attachment receptor angiotensin-converting enzyme 2 is decreased in crohn’s disease and regulated by microbial and inflammatory signaling. Gastroenterol. 2021;160(3):925–928 e924. doi:10.1053/j.gastro.2020.10.021. PMC7567698 33075345

[172] Zhang H, Li HB, Lyu JR, Lei XM, Li W, Wu G, Lyu J, Dai ZM. Specific ACE2 expression in small intestinal enterocytes may cause gastrointestinal symptoms and injury after 2019-nCov infection. Int J Infect Dis. 2020;96:19–24. doi:10.1016/j.ijid.2020.04.027. 32311451 PMC7165079

[173] Atkinson S, Sieffert E, Bihari D. A prospective, randomized, double-blind, controlled clinical trial of enteral immunonutrition in the critically ill. Guy’s hospital intensive care group. Crit Care Med. 1998;26(7):1164–1172. doi:10.1097/00003246-199807000-00013. 9671364

[174] Avontuur JA, Stam TC, Eggermont AMM, Braining HA, Jongen-Lavrencic M, van Amsterdam JGC. Effect of l-NAME, an inhibitor of nitric oxide synthesis, on plasma levels of IL-6, IL-8, TNFα and nitrite/nitrate in human septic shock. Intensive Care Med. 1998;24(7):673–679. doi:10.1007/s001340050643. 9722036

[175] Bertolini G, Iapichino G, Radrizzani D, Facchini R, Simini B, Bruzzone P, Zanforlin G, Tognoni G. Early enteral immunonutrition in patients with severe sepsis: results of an interim analysis of a randomized multicentre clinical trial. Intensive Care Med. 2003;29(5):834–840. doi:10.1007/s00134-003-1711-5. 12684745

[176] Buhrer C, Fischer HS, Wellmann S. Nutritional interventions to reduce rates of infection, necrotizing enterocolitis and mortality in very preterm infants. Pediatr Res. 2020;87(2):371–377. doi:10.1038/s41390-019-0630-2. 31645057

[177] Amin HJ, Zamora SA, McMillan DD, Fick GH, Butzner JD, Parsons HG, Scott RB. Arginine supplementation prevents necrotizing enterocolitis in the premature infant. J Pediatr. 2002;140(4):425–431. doi:10.1067/mpd.2002.123289. 12006956

[178] Shah PS, Shah VS, Kelly LE. Arginine supplementation for prevention of necrotising enterocolitis in preterm infants. Cochrane Database Syst Rev. 2017;4: CD004339. doi:10.1002/14651858.CD004339.pub4. PMC6478109 28399330

[179] Zhang Y, Gu F, Wang F, Zhang Y. Effects of early enteral nutrition on the gastrointestinal motility and intestinal mucosal barrier of patients with burn-induced invasive fungal infection. Pak J Med Sci. 2016;32(3):599–603. doi:10.12669/pjms.323.9717. 27375697 PMC4928406

[180] Nielsen AA, Nielsen JN, Grønbæk H, Eivindson M, Vind I, Munkholm P, Brandslund I, Hey H. Impact of enteral supplements enriched with ω–3 fatty acids and/or ω–6 fatty acids, arginine and ribonucleic acid compounds on leptin levels and nutritional status in active crohn’s disease treated with prednisolone. Digestion. 2007;75(1):10–16. doi:10.1159/000101560. 17429201

[181] Bologna C, Pone E. Clinical study on the efficacy and safety of arginine administered orally in association with other active ingredients for the prevention and treatment of sarcopenia in patients with COVID-19-related pneumonia, hospitalized in a sub-intensive care unit. Healthcare (Basel). 2022;10(1):162. doi:10.3390/healthcare10010162. 35052325 PMC8776134

[182] Tosato M, Calvani R, Picca A, Ciciarello F, Galluzzo V, Coelho-Júnior HJ, Di Giorgio A, Di Mario C, Gervasoni J, Gremese E, et al. Effects of l-Arginine plus vitamin C supplementation on physical performance, endothelial function, and persistent fatigue in adults with long COVID: a single-blind randomized controlled trial. Nutrients. 2022;14(23):4984. doi:10.3390/nu14234984. 36501014 PMC9738241

[183] Fiorentino G, Coppola A, Izzo R, Annunziata A, Bernardo M, Lombardi A, Trimarco V, Santulli G, Trimarco B. Effects of adding L-arginine orally to standard therapy in patients with COVID-19: a randomized, double-blind, placebo-controlled, parallel-group trial. Results of the first interim analysis. EClinicalMed. 2021;40:101125. doi:10.1016/j.eclinm.2021.101125. PMC8428476 34522871

[184] Muralidharan J, Kashyap SP, Jacob M, Ollapally A, Idiculla J, Raj JM, Thomas T, Kurpad AV. The effect of l-arginine supplementation on amelioration of oxygen support in severe COVID-19 pneumonia. Clin Nutr ESPEN. 2022;52:431–435. doi:10.1016/j.clnesp.2022.09.024. 36513483 PMC9511895

[185] Izzo R, Trimarco V, Mone P, Aloè T, Capra Marzani M, Diana A, Fazio G, Mallardo M, Maniscalco M, Marazzi G, et al. Combining L-Arginine with vitamin C improves long-COVID symptoms: the LINCOLN survey. Pharmacol Res. 2022;183:106360. doi:10.1016/j.phrs.2022.106360. 35868478 PMC9295384

[186] Di Renzo L, Gualtieri P, Pivari F, Soldati L, Attinà A, Leggeri C, Cinelli G, Tarsitano MG, Caparello G, Carrano E, et al. COVID-19: is there a role for immunonutrition in obese patient? J Transl Med. 2020;18(1):415. doi:10.1186/s12967-020-02594-4. 33160363 PMC7647877

[187] Saithong S, Worasilchai N, Saisorn W, Udompornpitak K, Bhunyakarnjanarat T, Chindamporn A, Tovichayathamrong P, Torvorapanit P, Chiewchengchol D, Chancharoenthana W, et al. Neutrophil extracellular traps in severe SARS-CoV-2 infection: a possible impact of LPS and (1→3)-β-D-glucan in blood from gut translocation. Cells. 2022;11(7):1103. doi:10.3390/cells11071103. 35406667 PMC8997739

[188] He Q, Shi Y, Xing H, Tang Q, Liu J, Li C, Zhang H, Zhang B, Zhang J, Chen X, et al. Modulating effect of xuanfei baidu granule on host metabolism and gut microbiome in rats. Front Pharmacol. 2022;13:922642. doi:10.3389/fphar.2022.922642. 36147334 PMC9486314

[189] Skendros P, Mitsios A, Chrysanthopoulou A, Mastellos DC, Metallidis S, Rafailidis P, Ntinopoulou M, Sertaridou E, Tsironidou V, Tsigalou C, et al. Complement and tissue factor–enriched neutrophil extracellular traps are key drivers in COVID-19 immunothrombosis. J Clin Invest. 2020;130(11):6151–6157. doi:10.1172/JCI141374. 32759504 PMC7598040

[190] Veras FP, Pontelli MC, Silva CM, Toller-Kawahisa JE, de Lima M, Nascimento DC, Schneider AH, Caetité D, Tavares LA, Paiva IM, et al. SARS-CoV-2–triggered neutrophil extracellular traps mediate COVID-19 pathology. J Exp Med. 2020;217(12). doi:10.1084/jem.20201129. PMC7488868 32926098

[191] Yang J, Tian C, Chen Y, Zhu C, Chi H, Li J. Obesity aggravates COVID-19: an updated systematic review and meta-analysis. J Med Virol. 2021;93(5):2662–2674. doi:10.1002/jmv.26677. 33200825 PMC7753795

[192] Yang Y, Wang L, Liu J, Fu S, Zhou L, Wang Y. Obesity or increased body mass index and the risk of severe outcomes in patients with COVID-19: a protocol for systematic review and meta-analysis. Med. 2022;101(1):e28499. doi:10.1097/MD.0000000000028499. PMC8735775 35029905

[193] Zhang X, Lewis AM, Moley JR, Brestoff JR. A systematic review and meta-analysis of obesity and COVID-19 outcomes. Sci Rep. 2021;11(1):7193. doi:10.1038/s41598-021-86694-1. 33785830 PMC8009961

[194] Marti ILAA, Reith W. Arginine-dependent immune responses. Cell Mol Life Sci. 2021;78(13):5303–5324. doi:10.1007/s00018-021-03828-4. 34037806 PMC8257534

[195] Strazar M, Temba GS, Vlamakis H, Kullaya VI, Lyamuya F, Mmbaga BT, Joosten LAB, van der Ven AJAM, Netea MG, de Mast Q, et al. Gut microbiome-mediated metabolism effects on immunity in rural and urban African populations. Nat Commun. 2021;12(1):4845. doi:10.1038/s41467-021-25213-2. 34381036 PMC8357928

[196] Kim YJ, Lee JY, Lee JJ, Jeon SM, Silwal P, Kim IS, Kim HJ, Park CR, Chung C, Han JE, et al. Arginine-mediated gut microbiome remodeling promotes host pulmonary immune defense against nontuberculous mycobacterial infection. Gut Microbes. 2022;14(1):2073132. doi:10.1080/19490976.2022.2073132. 35579969 PMC9116420

[197] Crowther RR, Qualls JE. Metabolic regulation of immune responses to mycobacterium tuberculosis: a spotlight on L-Arginine and L-Tryptophan metabolism. Front Immunol. 2020;11:628432. doi:10.3389/fimmu.2020.628432. 33633745 PMC7900187

[198] Bacher P, Hohnstein T, Beerbaum E, Röcker M, Blango MG, Kaufmann S, Röhmel J, Eschenhagen P, Grehn C, Seidel K, et al. Human anti-fungal Th17 immunity and pathology rely on cross-reactivity against Candida albicans. Cell. 2019;176(6):1340–1355 e1315. doi:10.1016/j.cell.2019.01.041. 30799037

[199] Shao TY, Ang WXG, Jiang TT, Huang FS, Andersen H, Kinder JM, Pham G, Burg AR, Ruff B, Gonzalez T, et al. Commensal Candida albicans positively calibrates systemic Th17 immunological responses. Cell Host Microbe. 2019;25(3):404–417 e406. doi:10.1016/j.chom.2019.02.004. 30870622 PMC6419754

[200] Al-Kuraishy HM, Al-Gareeb AI, Alexiou A, Batiha GE. COVID-19 and L-arginine supplementations: yet to find the missed key. Curr Protein Pept Sci. 2022;23(3):166–169. doi:10.2174/1389203723666220512104039. 35549865

[201] Arrieta MC, Arévalo A, Stiemsma L, Dimitriu P, Chico ME, Loor S, Vaca M, Boutin RCT, Morien E, Jin M, et al. Associations between infant fungal and bacterial dysbiosis and childhood atopic wheeze in a nonindustrialized setting. J Allergy Clin Immunol. 2018;142(2):424–434 e410. doi:10.1016/j.jaci.2017.08.041. 29241587 PMC6075469

[202] Fujimura KE, Sitarik AR, Havstad S, Lin DL, Levan S, Fadrosh D, Panzer AR, LaMere B, Rackaityte E, Lukacs NW, et al. Neonatal gut microbiota associates with childhood multisensitized atopy and T cell differentiation. Nat Med. 2016;22(10):1187–1191. doi:10.1038/nm.4176. 27618652 PMC5053876

[203] Ward TL, Dominguez-Bello MG, Heisel T, Al-Ghalith G, Knights D, Gale CA. Development of the human mycobiome over the first month of life and across body sites. mSystems. 2018;3(3). doi:10.1128/mSystems.00140-17. PMC5840654 29546248

[204] Hallen-Adams HE, Suhr MJ. Fungi in the healthy human gastrointestinal tract. Virulence. 2017;8(3):352–358. doi:10.1080/21505594.2016.1247140. 27736307 PMC5411236

[205] Li J, Chen D, Yu B, He J, Zheng P, Mao X, Yu J, Luo J, Tian G, Huang Z, et al. Fungi in gastrointestinal tracts of human and mice: from community to functions. Microb Ecol. 2018;75(4):821–829. doi:10.1007/s00248-017-1105-9. 29110065

[206] van Tilburg Bernardes E, Pettersen VK, Gutierrez MW, Laforest-Lapointe I, Jendzjowsky NG, Cavin JB, Vicentini FA, Keenan CM, Ramay HR, Samara J, et al. Intestinal fungi are causally implicated in microbiome assembly and immune development in mice. Nat Commun. 2020;11(1):2577. doi:10.1038/s41467-020-16431-1. 32444671 PMC7244730

[207] Richard ML, Sokol H. The gut mycobiota: insights into analysis, environmental interactions and role in gastrointestinal diseases. Nat Rev Gastroenterol Hepatol. 2019;16:331–345. doi:10.1038/s41575-019-0121-2. 30824884

[208] Kapitan M, Niemiec MJ, Steimle A, Frick JS, Jacobsen ID. Fungi as part of the microbiota and interactions with intestinal bacteria. Curr Top Microbiol Immunol. 2019;422:265–301. doi:10.1007/82_2018_117. 30062595

[209] Buonomo EL, Petri WA Jr. The microbiota and immune response during clostridium difficile infection. Anaerobe. 2016;41:79–84. doi:10.1016/j.anaerobe.2016.05.009. 27212111 PMC5050085

[210] Chao A, Vazquez JA. Fungal infections of the gastrointestinal tract. Gastroenterol Clin North Am. 2021;50(2):243–260. doi:10.1016/j.gtc.2021.02.009. 34024440

[211] Alonso-Monge R, Gresnigt MS, Roman E, Hube B, Pla J, Jarosz D. Candida albicans colonization of the gastrointestinal tract: a double-edged sword. PLoS Pathog. 2021;17(7):e1009710. doi:10.1371/journal.ppat.1009710. 34293071 PMC8297749

[212] Tso GHW, Reales-Calderon JA, Tan ASM, Sem X, Le GTT, Tan TG, Lai GC, Srinivasan KG, Yurieva M, Liao W, et al. Experimental evolution of a fungal pathogen into a gut symbiont. Sci. 2018;362(6414):589–595. doi:10.1126/science.aat0537. 30385579

[213] Doron I, Leonardi I, Li XV, Fiers WD, Semon A, Bialt-DeCelie M, Migaud M, Gao IH, Lin WY, Kusakabe T, et al. Human gut mycobiota tune immunity via CARD9-dependent induction of anti-fungal IgG antibodies. Cell. 2021;184(4):1017–1031.e14. doi:10.1016/j.cell.2021.01.016. 33548172 PMC7936855

[214] Uppuluri P, Lin L, Alqarihi A, Luo G, Youssef EG, Alkhazraji S, Yount NY, Ibrahim BA, Bolaris MA, Edwards JE, et al. The Hyr1 protein from the fungus Candida albicans is a cross kingdom immunotherapeutic target for acinetobacter bacterial infection. PLoS Pathog. 2018;14(5):e1007056. doi:10.1371/journal.ppat.1007056. 29746596 PMC5963808

[215] Underhill DM, Braun J. Fungal microbiome in inflammatory bowel disease: a critical assessment. J Clin Invest. 2022;132(5). doi:10.1172/JCI155786. PMC8884899 35229726

[216] Chehoud C, Albenberg LG, Judge C, Hoffmann C, Grunberg S, Bittinger K, Baldassano RN, Lewis JD, Bushman FD, Wu GD, et al. Fungal signature in the gut microbiota of pediatric patients with inflammatory bowel disease. Inflamm Bowel Dis. 2015;21(8):1948–1956. doi:10.1097/MIB.0000000000000454. 26083617 PMC4509842

[217] Lewis JD, Chen E, Baldassano R, Otley A, Griffiths A, Lee D, Bittinger K, Bailey A, Friedman E, Hoffmann C, et al. Inflammation, antibiotics, and diet as environmental stressors of the gut microbiome in pediatric crohn’s disease. Cell Host Microbe. 2015;18(4):489–500. doi:10.1016/j.chom.2015.09.008. 26468751 PMC4633303

[218] Sokol H, Leducq V, Aschard H, Pham HP, Jegou S, Landman C, Cohen D, Liguori G, Bourrier A, Nion-Larmurier I, et al. Fungal microbiota dysbiosis in IBD. Gut. 2017;66(6):1039–1048. doi:10.1136/gutjnl-2015-310746. 26843508 PMC5532459

[219] Begum N, Lee S, Portlock TJ, Pellon A, Nasab SDS, Nielsen J, Uhlen M, Moyes DL, Shoaie S. Integrative functional analysis uncovers metabolic differences between Candida species. Commun Biol. 2022;5(1):1013. doi:10.1038/s42003-022-03955-z. 36163459 PMC9512779

[220] Jimenez-Lopez C, Collette JR, Brothers KM, Shepardson KM, Cramer RA, Wheeler RT, Lorenz MC. Candida albicans induces arginine biosynthetic genes in response to host-derived reactive oxygen species. Eukaryot Cell. 2013;12(1):91–100. doi:10.1128/EC.00290-12. 23143683 PMC3535846

[221] Ghosh S, Navarathna DHMLP, Roberts DD, Cooper JT, Atkin AL, Petro TM, Nickerson KW. Arginine-induced germ tube formation in Candida albicans is essential for escape from murine macrophage line RAW 264.7. Infect Immun. 2009;77(4):1596–1605. doi:10.1128/IAI.01452-08. 19188358 PMC2663133

[222] Wagener J, MacCallum DM, Brown GD, Gow NA, Doering TL. Candida albicans chitin increases Arginase-1 activity in human macrophages, with an impact on macrophage antimicrobial functions. mBio. 2017;8(1). doi:10.1128/mBio.01820-16. PMC5263244 28119468

[223] Nishimura A, Nakagami K, Kan K, Morita F, Takagi H. Arginine inhibits saccharomyces cerevisiae biofilm formation by inducing endocytosis of the arginine transporter Can1. Biosci Biotechnol Biochem. 2022;86(9):1300–1307. doi:10.1093/bbb/zbac094. 35749478

[224] Ma N, Tian Y, Wu Y, Ma X. Contributions of the interaction between dietary protein and gut microbiota to intestinal health. Curr Protein Pept Sci. 2017;18(8):795–808. doi:10.2174/1389203718666170216153505. 28215168

[225] Spadoni I, Fornasa G, Rescigno M. Organ-specific protection mediated by cooperation between vascular and epithelial barriers. Nat Rev Immunol. 2017;17(12):761–773. doi:10.1038/nri.2017.100. 28869253

[226] Spadoni I, Zagato E, Bertocchi A, Paolinelli R, Hot E, Di Sabatino A, Caprioli F, Bottiglieri L, Oldani A, Viale G, et al. A gut-vascular barrier controls the systemic dissemination of bacteria. Sci. 2015;350(6262):830–834. doi:10.1126/science.aad0135. 26564856

[227] Martel J, Chang SH, Ko YF, Hwang TL, Young JD, Ojcius DM. Gut barrier disruption and chronic disease. Trends Endocrinol Metab. 2022;33(4):247–265. doi:10.1016/j.tem.2022.01.002. 35151560

[228] Wu M, Xiao H, Shao F, Tan B, Hu S. Arginine accelerates intestinal health through cytokines and intestinal microbiota. Int Immunopharmacol. 2020;81:106029. doi:10.1016/j.intimp.2019.106029. 31757675

[229] Ren W, Chen S, Yin J, Duan J, Li T, Liu G, Feng Z, Tan B, Yin Y, Wu G, et al. Dietary arginine supplementation of mice alters the microbial population and activates intestinal innate immunity. J Nutr. 2014;144(6):988–995. doi:10.3945/jn.114.192120. 24670969

[230] Monticelli LA, Buck MD, Flamar AL, Saenz SA, Tait Wojno ED, Yudanin NA, Osborne LC, Hepworth MR, Tran SV, Rodewald HR, et al. Arginase 1 is an innate lymphoid-cell-intrinsic metabolic checkpoint controlling type 2 inflammation. Nat Immunol. 2016;17(6):656–665. doi:10.1038/ni.3421. 27043409 PMC4873382

[231] Holzapfel W, Arini A, Aeschbacher M, Coppolecchia R, Pot B. Enterococcus faecium SF68 as a model for efficacy and safety evaluation of pharmaceutical probiotics. Benef Microbes. 2018;9(3):375–388. doi:10.3920/BM2017.0148. 29633645

[232] Wunderlich PF, Braun L, Fumagalli I, D’Apuzzo V, Heim F, Karly M, Lodi R, Politta G, Vonbank F, Zeltner L, et al. Double-blind report on the efficacy of lactic acid-producing enterococcus SF68 in the prevention of antibiotic-associated diarrhoea and in the treatment of acute diarrhoea. J Int Med Res. 1989;17(4):333–338. doi:10.1177/030006058901700405. 2676650

[233] Tian J, Utter DR, Cen L, Dong PT, Shi W, Bor B, Qin M, McLean JS, He X. Acquisition of the arginine deiminase system benefits epiparasitic saccharibacteria and their host bacteria in a mammalian niche environment. Proc Natl Acad Sci USA. 2022;119(2). doi:10.1073/pnas.2114909119. PMC8764695 34992141

[234] Wei Z, Oh J, Flavell RA, Crawford JM. LACC1 bridges NOS2 and polyamine metabolism in inflammatory macrophages. Nature. 2022;609(7926):348–353. doi:10.1038/s41586-022-05111-3. 35978195 PMC9813773

[235] Shiloh MU, MacMicking JD, Nicholson S, Brause JE, Potter S, Marino M, Fang F, Dinauer M, Nathan C. Phenotype of mice and macrophages deficient in both phagocyte oxidase and inducible nitric oxide synthase. Immunity. 1999;10(1):29–38. doi:10.1016/s1074-7613(00)80004-7. 10023768

[236] Vazquez-Torres A, Baumler AJ. Nitrate, nitrite and nitric oxide reductases: from the last universal common ancestor to modern bacterial pathogens. Curr Opin Microbiol. 2016;29:1–8. doi:10.1016/j.mib.2015.09.002. 26426528 PMC4930242

[237] Winter SE, Winter MG, Xavier MN, Thiennimitr P, Poon V, Keestra AM, Laughlin RC, Gomez G, Wu J, Lawhon SD, et al. Host-derived nitrate boosts growth of E. coli in the inflamed gut. Science. 2013;339(6120):708–711. doi:10.1126/science.1232467. 23393266 PMC4004111

[238] Liou MJ, Miller BM, Litvak Y, Nguyen H, Natwick DE, Savage HP, Rixon JA, Mahan SP, Hiyoshi H, Rogers AWL, et al. Host cells subdivide nutrient niches into discrete biogeographical microhabitats for gut microbes. Cell Host & Microbe. 2022;30(6):836–847 e836. doi:10.1016/j.chom.2022.04.012. 35568027 PMC9187619

[239] Walker MY, Pratap S, Southerland JH, Farmer-Dixon CM, Lakshmyya K, Gangula PR. Role of oral and gut microbiome in nitric oxide-mediated colon motility. Nitric Oxide. 2018;73:81–88. doi:10.1016/j.niox.2017.06.003. 28602746 PMC6104390

[240] Roediger WE. Review article: nitric oxide from dysbiotic bacterial respiration of nitrate in the pathogenesis and as a target for therapy of ulcerative colitis. Aliment Pharmacol Ther. 2008;27(7):531–541. doi:10.1111/j.1365-2036.2008.03612.x. 18194497

[241] Moretti C, Zhuge Z, Zhang G, Haworth SM, Paulo LL, Guimarães DD, Cruz JC, Montenegro MF, Cordero-Herrera I, Braga VA, et al. The obligatory role of host microbiota in bioactivation of dietary nitrate. Free Radic Biol Med. 2019;145:342–348. doi:10.1016/j.freeradbiomed.2019.10.003. 31600544

[242] Knox NC, Forbes JD, Van Domselaar G, Bernstein CN. The gut microbiome as a target for IBD treatment: are we there yet? Curr Treat Options Gastroenterol. 2019;17(1):115–126. doi:10.1007/s11938-019-00221-w. 30661163

[243] Nüse B, Mattner J. L-arginine as a novel target for clinical intervention in inflammatory bowel disease. Expl Immunol. 2021;1:80–89. doi:10.37349/ei.2021.00008.

[244] Berer K, Gerdes LA, Cekanaviciute E, Jia X, Xiao L, Xia Z, Liu C, Klotz L, Stauffer U, Baranzini SE, et al. Gut microbiota from multiple sclerosis patients enables spontaneous autoimmune encephalomyelitis in mice. Proc Natl Acad Sci USA. 2017;114(40):10719–10724. doi:10.1073/pnas.1711233114. 28893994 PMC5635914

[245] Cekanaviciute E, Yoo BB, Runia TF, Debelius JW, Singh S, Nelson CA, Kanner R, Bencosme Y, Lee YK, Hauser SL, et al. Gut bacteria from multiple sclerosis patients modulate human T cells and exacerbate symptoms in mouse models. Proc Natl Acad Sci USA. 2017;114(40):10713–10718. doi:10.1073/pnas.1711235114. 28893978 PMC5635915

[246] Zhang X, Zhang D, Jia H, Feng Q, Wang D, Liang D, Wu X, Li J, Tang L, Li Y, et al. The oral and gut microbiomes are perturbed in rheumatoid arthritis and partly normalized after treatment. Nat Med. 2015;21(8):895–905. doi:10.1038/nm.3914. 26214836

[247] Zhu Q, Hou Q, Huang S, Ou Q, Huo D, Vázquez-Baeza Y, Cen C, Cantu V, Estaki M, Chang H, et al. Compositional and genetic alterations in graves’ disease gut microbiome reveal specific diagnostic biomarkers. Isme J. 2021;15(11):3399–3411. doi:10.1038/s41396-021-01016-7. 34079079 PMC8528855

[248] Hou Q, Dong Y, Huang J, Liao C, Lei J, Wang Y, Lai Y, Bian Y, He Y, Sun J, et al. Exogenous L-arginine increases intestinal stem cell function through CD90+ stromal cells producing mTORC1-induced Wnt2b. Commun Biol. 2020;3(1):611. doi:10.1038/s42003-020-01347-9. 33097830 PMC7584578

[249] Hou Q, Dong Y, Yu Q, Wang B, Le S, Guo Y, Zhang B. Regulation of the paneth cell niche by exogenous L-arginine couples the intestinal stem cell function. Faseb J. 2020;34(8):10299–10315. doi:10.1096/fj.201902573RR. 32725957

[250] Longley DB, Harkin DP, Johnston PG. 5-fluorouracil: mechanisms of action and clinical strategies. Nat Rev Cancer. 2003;3(5):330–338. doi:10.1038/nrc1074. 12724731

[251] Li JM, Yang DC, Oldham J, Linderholm A, Zhang J, Liu J, Kenyon NJ, Chen CH. Therapeutic targeting of argininosuccinate synthase 1 (ASS1)-deficient pulmonary fibrosis. Molecular Therapy. 2021;29(4):1487–1500. doi:10.1016/j.ymthe.2021.01.028. 33508432 PMC8058484

[252] McNeal CJ, Meininger CJ, Reddy D, Wilborn CD, Wu G. Safety and effectiveness of Arginine in adults. J Nutr. 2016;146(12):2587S–2593S. doi:10.3945/jn.116.234740. 27934649

[253] Rosenthal MD, Carrott PW, Patel J, Kiraly L, Martindale RG. Parenteral or enteral arginine supplementation safety and efficacy. J Nutr. 2016;146(12):2594S–2600S. doi:10.3945/jn.115.228544. 27934650

[254] Lopez LR, Bleich RM, Arthur JC. Microbiota effects on carcinogenesis: initiation, promotion, and progression. Annu Rev Med. 2021;72(1):243–261. doi:10.1146/annurev-med-080719-091604. 33052764 PMC10202020

[255] Stettner N, Rosen C, Bernshtein B, Gur-Cohen S, Frug J, Silberman A, Sarver A, Carmel-Neiderman NN, Eilam R, Biton I, et al. Induction of nitric-oxide metabolism in enterocytes alleviates colitis and inflammation-associated colon cancer. Cell Rep. 2018;23(7):1962–1976. doi:10.1016/j.celrep.2018.04.053. 29768197 PMC5976577

[256] Huang HL, Chen WC, Hsu HP, Cho CY, Hung YH, Wang CY, Lai MD. Silencing of argininosuccinate lyase inhibits colorectal cancer formation. Oncology Reports. 2017;37(1):163–170. doi:10.3892/or.2016.5221. 27840980

[257] van der Meer JHM, de Boer RJ, Meijer BJ, Smit WL, Vermeulen JLM, Meisner S, van Roest M, Koelink PJ, Dekker E, Hakvoort TBM, et al. Epithelial argininosuccinate synthetase is dispensable for intestinal regeneration and tumorigenesis. Cell Death Dis. 2021;12(10):897. doi:10.1038/s41419-021-04173-x. 34599156 PMC8486827

[258] Markey L, Shaban L, Green ER, Lemon KP, Mecsas J, Kumamoto CA. Pre-colonization with the commensal fungus Candida albicans reduces murine susceptibility to clostridium difficile infection. Gut Microbes. 2018;9:497–509. doi:10.1080/19490976.2018.1465158. 29667487 PMC6287688

[259] Panpetch W, Hiengrach P, Nilgate S, Tumwasorn S, Somboonna N, Wilantho A, Chatthanathon P, Prueksapanich P, Leelahavanichkul A. Additional Candida albicans administration enhances the severity of dextran sulfate solution induced colitis mouse model through leaky gut-enhanced systemic inflammation and gut-dysbiosis but attenuated by Lactobacillus rhamnosus L34. Gut Microbes. 2020;11(3):465–480. doi:10.1080/19490976.2019.1662712. 31530137 PMC7527076

[260] Bertolini M, Ranjan A, Thompson A, Diaz PI, Sobue T, Maas K, Dongari-Bagtzoglou A. Candida albicans induces mucosal bacterial dysbiosis that promotes invasive infection. PLoS Pathog. 2019;15(4):e1007717. doi:10.1371/journal.ppat.1007717. 31009520 PMC6497318

[261] Rosshart SP, Herz J, Vassallo BG, Hunter A, Wall MK, Badger JH, McCulloch JA, Anastasakis DG, Sarshad AA, Leonardi I, et al. Laboratory mice born to wild mice have natural microbiota and model human immune responses. Science. 2019;365(6452). doi:10.1126/science.aaw4361. PMC7377314 31371577

[262] Lin JD, Devlin JC, Yeung F, McCauley C, Leung JM, Chen Y-H, Cronkite A, Hansen C, Drake-Dunn C, Ruggles KV, et al. Rewilding Nod2 and Atg16l1 mutant mice uncovers genetic and environmental contributions to microbial responses and immune cell composition. Cell Host & Microbe. 2020;27(5):830–840 e834. doi:10.1016/j.chom.2020.03.001. 32209431 PMC7228860

[263] Yeung F, Chen YH, Lin JD, Leung JM, McCauley C, Devlin JC, Hansen C, Cronkite A, Stephens Z, Drake-Dunn C, et al. Altered immunity of laboratory mice in the natural environment is associated with fungal colonization. Cell Host & Microbe. 2020;27(5):809–822 e806. doi:10.1016/j.chom.2020.02.015. 32209432 PMC7276265

[264] Lapiere A, Richard ML. Bacterial-fungal metabolic interactions within the microbiota and their potential relevance in human health and disease: a short review. Gut Microbes. 2022;14(1):2105610. doi:10.1080/19490976.2022.2105610. 35903007 PMC9341359

[265] Carbajosa S, Rodríguez-Angulo HO, Gea S, Chillón-Marinas C, Poveda C, Maza MC, Colombet D, Fresno M, Gironès N. L-arginine supplementation reduces mortality and improves disease outcome in mice infected with trypanosoma cruzi. PLoS Negl Trop Dis. 2018;12(1):e0006179. doi:10.1371/journal.pntd.0006179. 29337988 PMC5786330

[266] Sakanyan V, Petrosyan P, Lecocq M, Boyen A, Legrain C, Demarez M, Hallet JN, Glansdorff N. Genes and enzymes of the acetyl cycle of arginine biosynthesis in corynebacterium glutamicum: enzyme evolution in the early steps of the arginine pathway. Microbiol. 1996;142(1):99–108. doi:10.1099/13500872-142-1-99. 8581175

